# Reusable SnS_2_‑Based Cotton Fabric Composites for Efficient Decontamination of Water from Lead Ions under Continuous Flow Conditions

**DOI:** 10.1021/acs.langmuir.5c01540

**Published:** 2025-05-28

**Authors:** Vasiliki I. Karagianni, Efthymia Toti, Christos Dimitriou, Yiannis Deligiannakis, Alexios P. Douvalis, Manolis J. Manos

**Affiliations:** † Department of Chemistry, 37796 University of Ioannina, Ioannina GR-45110, Greece; ‡ Department of Physics, 37796 University of Ioannina, Ioannina GR-45110, Greece

## Abstract

Lead is a toxic heavy metal that pollutes the environment and accumulates in the human body, causing many severe health issues. Metal sulfides have emerged as promising sorbents for rapidly decontaminating Pb^2+^-containing wastewater, showing exceptional sorption kinetics, capacities, and selectivity against common competitive ionic species. In this study, we present modified SnS_2_ phases, namely, SnS_2_(DMA)_0.7_(H_2_O)_0.3_ (**SnS**_**2**_ **/DMA**, DMA = dimethylamine) and Sn_1–*x*_S_2_·*y*H_2_O (**SnS**_**2**_ **/acid**), which demonstrated efficient removal of Pb^2+^ ions from aqueous solutions. Both materials exhibited fast kinetics (≤4 min), high sorption capacities (838.0 mg g^–1^ for **SnS**_**2**_ **/DMA** and 190.0 mg g^–1^ for **SnS**_**2**_ **/acid**), remarkable selectivity toward Pb^2+^ over several competing cations and in various pH values, because of strong Pb–S covalent interactions. Aiming for practical wastewater treatment, we immobilized **SnS**_**2**_ **/DMA** and **SnS**_**2**_ **/acid** on cotton fabrics, marking this as the initial application of metal sulfides immobilized onto cotton substrates. The metal sulfide-fabric composites were utilized to remove Pb^2+^ under continuous flow conditions, showing significant Pb^2+^ sorption properties. Significantly, the metal sulfide-based composites can be regenerated and reused over several Pb^2+^ sorption cycles. This feature, demonstrated for the first time in metal sulfide materials, constitutes a breakthrough for this class of sorbents.

## Graphical abstract

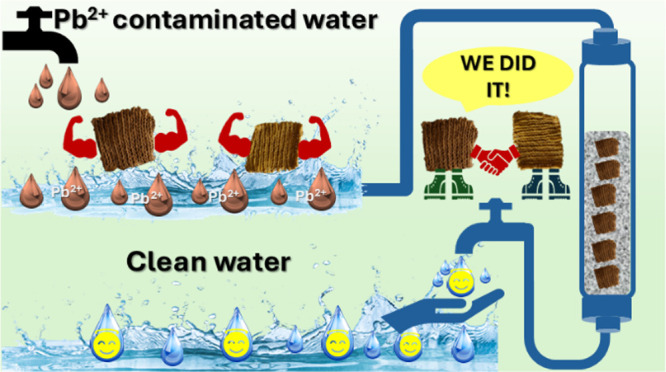

## 1 Introduction

The accumulation of heavy metals in the environment is a significant issue due to its repercussions on human health and the ecosystem. Heavy metal pollution derives from expanding industrial activities, mining, lead-acid battery manufacturing or recycling, industrial waste disposal, paint production, excessive fertilizer use, chrome plating, leather tanning, etc.^1,2^ Among the common pollutants, Pb^2+^ is very harmful, toxic, and carcinogenic, even in ppb (parts per billion) concentrations.^3^ The World Health Organization has defined tap water’s maximum acceptable total lead limit as 10 μg L^–1^. At the same time, in 2021, the EU decided to reduce the drinking water limit to 5 μg L^–1^, a goal that must be achieved by 2036.^4^

With its sizable atomic mass, lead is one of the most abundant heavy metals. Lead has become a global issue because of its toxicity to humans, animals, and the environment.^5^ In ancient times, Pb^2+^ was commonly used for making water pipes, coins, and various tools^6,7^ due to its availability and properties.^8^ Lead poisoning poses significant health risks, particularly impacting neurological development in children,^9^ as well as a range of physical health problems, including kidney impairment,^10^ cardiovascular issues,^11^ and gastrointestinal symptoms.^12^ Therefore, capturing and removing Pb^2+^ from industrial sewage is crucial before it is released into the water system.

Ion-exchange,^13^ chemical precipitation,^14^ membrane filtration^15^ and electrochemical techniques^16^ have been utilized to decrease Pb^2+^ levels in wastewater efficiently. Although each of these methods has advantages, there are significant drawbacks, such as high operating expenses, production of sludge, low selectivity for lead ions, and the methods’ complexity. On the other hand, sorption has captured the researchers’ interest due to its low cost, high sensitivity, regenerative ability, and cost-effectiveness.^17^

Metal sulfides (MSs) such as MoS_2_, SnS_2_, K_2*x*_Mn_*x*_Sn_3–*x*_S_6_ (KMS-1),^18^ K_2*x*_Mg_*x*_Sn_3–*x*_S_6_ (KMS-2)^19^ and H_2*x*_Mn_*x*_Sn_3–*x*_S_6_ (LHMS)^20^ have been employed extensively in water remediation, as their unique structural features offer many advantages.^21–24^ Based on the Lewis acid–base theory principles, MSs are up-and-coming candidates for decontaminating water from heavy metals because the soft-acidic metal ions create strong covalent bonds with soft base S^2–^ ligands.^25^ Among MSs, tin disulfide (SnS_2_) has been widely used for environmental purposes, such as the degradation of organic dyes,^26^ adsorption,^27^ reduction of Cr^6+^ ^28,29^ production of H_2,_ ^30^ and CO_2_ reduction.^31,32^ SnS_2_ is a layered MS semiconductor (*n*-type IV–VI) with bandgap energy in the 2.2–2.4 range.^33^ It exhibits a CdI_2_-type hexagonal crystal structure, where covalent bonds bind the intralayer atoms while van der Waals forces keep the individual layers together. This kind of layered structure allows the accommodation of ions (Li^2+^ ^34^) or molecules (amines^35^), resulting in the modification of the physical properties of the pristine material.

Despite their promising properties for heavy metal ion capture, MSs are not reusable, which may restrict their practical applications in wastewater treatment.^13,36^ Furthermore, MSs tested for heavy metal ion removal are usually tiny crystals or microcrystalline powder, making their practical applications in environmental remediation challenging and likely not feasible.^18,19^ One of the main difficulties is retrieving powdered sorbents from large water bodies and the risk of environmental pollution and increased turbidity, which can harm the ecosystem. One possible way to address these issues is by immobilizing sorbents onto bulk substrates like cotton textiles. These fabric composites can be submerged in water to capture harmful contaminants and easily retrieved afterward.^37^ MSs immobilized on bulk substrates could also be utilized as filters for wastewater decontamination under continuous flow, thus expanding their applications toward water treatment and reuse.

In this work, we report the synthesis and characterization of new modified SnS_2_ phases, SnS_2_(DMA)_0.7_(H_2_O)_0.3_ (**SnS**_**2**_ **/DMA**, DMA = Dimethylamine) and Sn_1–*x*_S_2_·*y*H_2_O (**SnS**_**2**_ **/acid**). These materials indicate exceptional Pb^2+^ sorption properties combined with fast kinetics and high removal capacities at high (100 ppm) or lower (1 ppm) concentrations of Pb^2+^, even amidst various competitive ions (Na^+^, Ca^2+^, and Mg^2+^). Both materials were immobilized on cotton fabrics using poly­(methyl methacrylate) (PMMA) as a binder. Notably, these composite materials could decontaminate relatively large amounts of wastewater simulant under continuous flow and are reusable. The reusability of these SnS_2_-based cotton filters, which has not been reported for metal sulfide materials, combined with the relatively low cost of the metal sulfide materials and the abundance of cotton, make them particularly attractive for applications in wastewater purification.

## 2 Experimental Section

### 2.1 Synthesis of SnS_2_(DMA)_0.7_(H_2_O)_0.3_ (SnS_2_/DMA)

Sn (0.2 g, 1.68 mmol) and S powder (0.162 g, 5.04 mmol) were combined in a 23 mL Teflon cup with a mixture of 0.6 mL of an aqueous solution (40% w/w) of dimethylamine (1.78 g, 39.5 mmol) and 2 mL of deionized water. The cup was covered with a lid and sealed inside a stainless-steel Parr autoclave. The autoclave was maintained at 180 °C in a preheated oven for 4 days under autogenous pressure. After 4 days, the autoclave was left to cool to room temperature. The resulting orange powder was centrifugated, washed repeatedly with water (x2) and acetone (x1), and was left to dry overnight in an oven at 60 °C (Yield = 0.179 g).

### 2.2 Synthesis of Sn_1–*x*_S_2_·*y*H_2_O (SnS_2_/Acid)

300 mg (1.365 mmol) of **SnS**_**2**_ **/DMA** and 30 mL of HCl (1 M) were added in a conical flask and the mixture was left stirring overnight. The resulting product was centrifugated, washed repeatedly with water (x4) and acetone (x1), and was left to dry overnight in an oven at 60 °C. This procedure was repeated twice (Yield = 0.243 g).

### 2.3 Solid-State Synthesis of SnS_2_

0.5 g (4.21 mmol) of Sn powder and 0.283 g (8.84 mmol) of S powder were mixed using a mortar and pestle, and the mixture was put in a pellet press under 9 Tons pressure for about 15 min. Then, the obtained pellet was smashed into three smaller pieces and sealed in a quartz ampule under vacuum (10^–3^ Torr), which was annealed at 500 °C for 24 h (Yield = 0.52 g).

### 2.4 Immobilization of MS onto the Cotton Fabric

Ten mg of PMMA were dissolved in 5 mL of acetone, followed by ultrasonication for 15 min. Next, 10 mg of MS were introduced into the PMMA solution and stirred at room temperature for 2–3 h until a homogeneous suspension was obtained. Following this, a piece of cotton fabric with a tetragonal shape (and an area of 1 cm^2^) was submerged into the suspension briefly while stirring and left to dry in the air. The process of immersing and drying was repeated several times until the entire suspension was consumed. To promote stronger adhesion of the MS to the cotton fabric, the composite was left to dry at 60 °C overnight.

### 2.5 Determination of the MS Content onto the PMMA@Cotton Fabric Composites

MS particles can be easily extracted from MS-PMMA@Cotton fabric composites due to the high solubility of PMMA in acetone, allowing for precise quantification of MS content. As acetone dissolves PMMA, the MS particles can no longer adhere to the fabric. The composites were immersed in 10 mL of acetone, sonicated, and stirred for ∼30 min. The fabric pieces were removed from the suspension. The latter was centrifuged to isolate the released MS particles. The extracted MS was dried at 60 °C for 1 h. The calculated masses for the six MS-PMMA@Cotton Fabrics used in each column were 16.6 and 17.1 mg for **SnS**_**2**_ **/DMA** and **SnS**_**2**_ **/acid,** respectively.

## 3 Results and Discussion

### 3.1 Synthesis and Structural Characterization

The solvothermal method is frequently used to create ion-intercalated metal sulfides.^38–40^ Intercalation of neutral molecules, such as amines, in SnS_2_ typically requires a two-step process: (a) synthesis of SnS_2_ (whether in crystals or bulk form) and (b) the subsequent intercalation of amines, often achieved by stirring and heating SnS_2_ in the amine solution or by repeating a solvothermal reaction.^41,42^ As a result of exploratory synthesis, aiming at new metal chalcogenide materials, we have synthesized new SnS_2_-based phases, namely **SnS**_**2**_ **/DMA** and **SnS**_**2**_ **/acid. SnS**_**2**_ **/DMA** was synthesized via a one-step solvothermal reaction involving tin (Sn) metal and sulfur (S) powder in a diluted aqueous dimethylamine solution at 180 °C. **SnS**_**2**_ **/acid** is produced by the acid-induced conversion of **SnS**_**2**_ **/DMA**, which removes dimethylamine from the interlayer spacing.

Unfortunately, we could not obtain single crystals of **SnS**_**2**_ **/DMA** for precise structural determination via single-crystal X-ray crystallography. Therefore, we used powder X-ray diffraction (PXRD) to identify its structural characteristics. Unit cell indexing indicated that **SnS**_**2**_ **/DMA** crystallizes in the hexagonal/trigonal crystal system and *P*3̅*m*1 space group. Le Bail analysis was conducted using TOPAS,^43^ and the results were quite satisfactory (Figure 1A). The refined unit cell parameters were determined to be *a* = 3.545(2) Å, *c* = 9.51(6) Å and *V* = 103.6(1) Å^3^ (space group *P*3̅*m*1). The PXRD data revealed a significant expansion of the interlayer spacing in **SnS**_**2**_ **/DMA** material, being *d* = 9.53 Å, compared to that (*d* = 5.89 Å) for the nonintercalated SnS_2_ compound. Such an expansion can be justified by the insertion of dimethylamine in the interlayer space. Specifically, subtracting the covalent radius of S atoms (2 × 1.05 Å) from the distance (6.9 Å) between the S atoms from adjacent layers of **SnS**_**2**_ **/DMA**, the available interlayer spacing is 4.8 Å. Considering that dimethylamine’s size is about 4.42 Å (distance between the farthest H atoms is 3.8 Å, plus the H covalent radius being 2 × 0.31 Å), it can be confirmed that the space between the layers of **SnS**_**2**_ **/DMA** can fit dimethylamine molecules (Figure 1B,C). PXRD measurements (Figure S1) also showed that **SnS**_**2**_ **/acid** is a layered material, isostructural with SnS_2_ (space group: *P*3̅*m*1, *a* = 3.65 Å, *c* = 5.89 Å, *V* = 67.86 Å^3^). Le Bail refinement was also performed on **SnS**_**2**_ **/acid** (Figure 1D), resulting in refined unit cell parameters: *a* = 3.60(2), *c* = 6.00(4) Å and *V* = 67.2(1) Å^3^ (space group *P*3̅*m*1).

**Figure 1:**
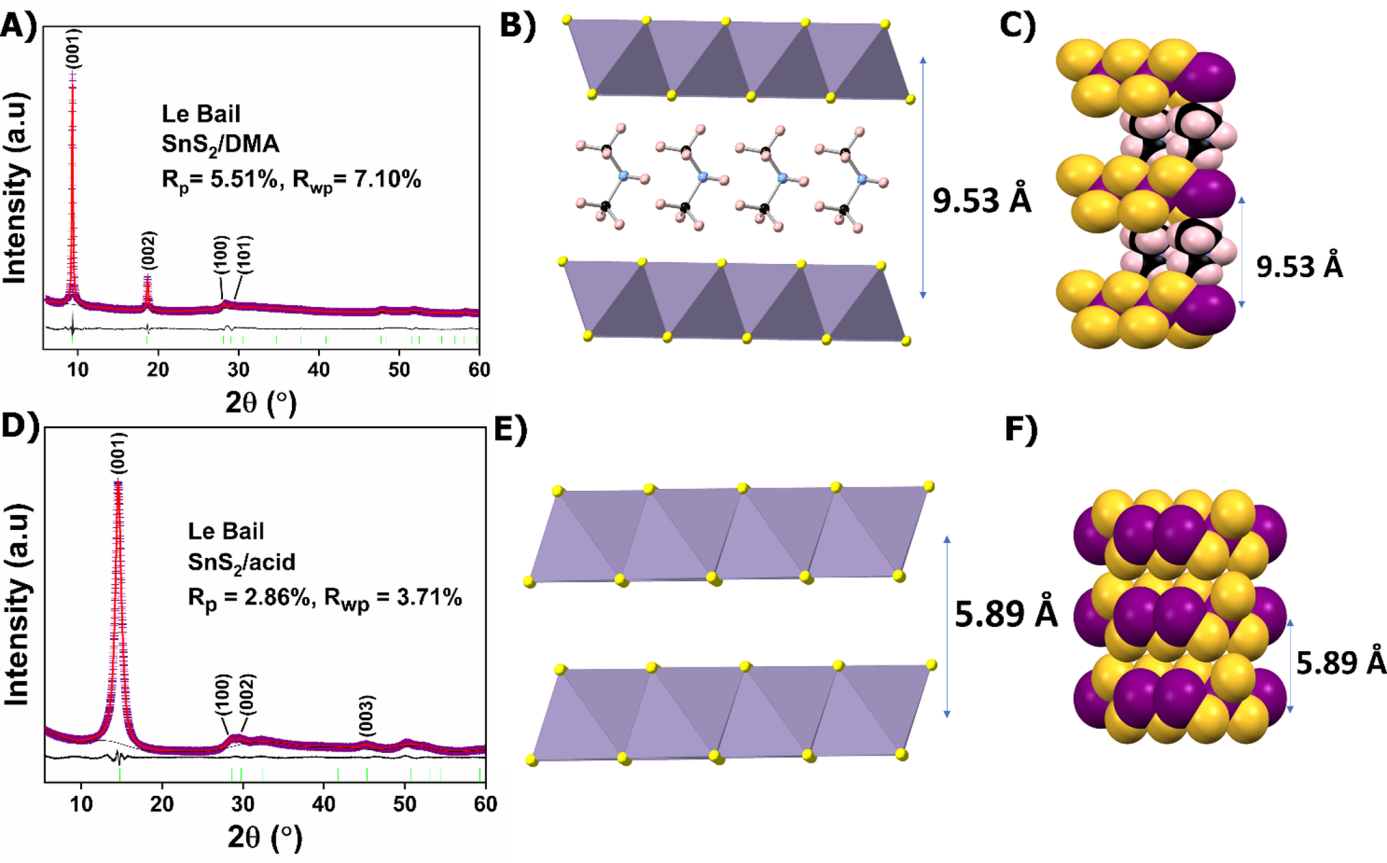
(A) Le Bail plot of **SnS**_**2**_ **/DMA**, Structural models of **SnS**_**2**_ **/DMA** with (B) polyhedral representation of the layers viewed along the *c*-axis, (C) space-filling model of **SnS**_**2**_ **/DMA** (element color coding: S, yellow; Sn, purple; C, black; Ν, blue; and Η, light pink), (D) Le Bail plot of **SnS**_**2**_ **/acid**, Structural models of SnS_2_ with (E) polyhedral representation of the layers viewed along the *c*-axis, (F) space-filling model of **SnS**_**2**_ (element color coding: S, yellow; Sn, purple). In the Le Bail plots, violet crosses represent experimental points, the red line corresponds to the calculated pattern, the black line shows the difference pattern (exp.–calc.), and green bars indicate the Bragg positions.

UV–vis Spectroscopy revealed that **SnS**_**2**_ **/DMA** and **SnS**_**2**_ **/acid** are wide-bandgap semiconductors with a band gap energy of 2.23 and 1.86 eV, respectively. The band gap of **SnS**_**2**_ **/DMA** is relatively close to that of nonintercalated **SnS**_**2**_ (2.20 eV), which agrees with the similar colors of these materials. In contrast, the darker **SnS**_**2**_ **/acid** has a much lower band gap (Figure 2B,E). The presence of defects in the structure of **SnS**_**2**_ **/acid** (see below) likely introduces electronic states within the band gap, enhancing visible light absorption and altering the material’s optical properties.^44,45^

**Figure 2:**
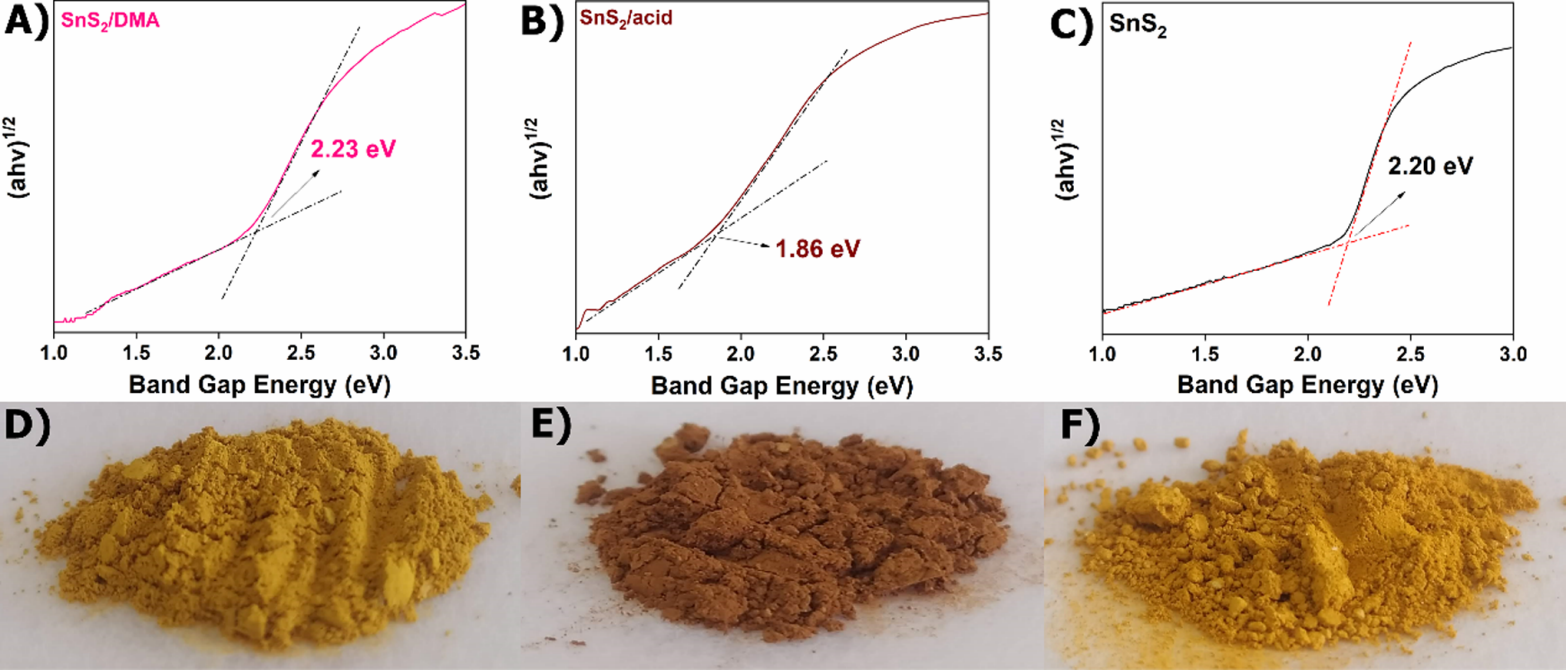
Tauc plot of (A) **SnS**_**2**_ **/DMA**, (B) **SnS**_**2**_ **/acid**, (C) **SnS**_**2**_, and images of the color of each solid (D–F), respectively. The linear part of the plot is extrapolated to the *x*-axis.

The structural stability of **SnS**_**2**_ **/DMA** was evaluated versus temperature by using Variable-Temperature PXRD (VT-PXRD). The VT-PXRD data revealed that the structure of **SnS**_**2**_ **/DMA** remains intact within the temperature range of 50 to 100 °C (Figure S2). However, from 150 to 300 °C, a new diffraction peak is observed at ∼14.9° (Figure S3), ascribed to the SnS_2_ phase. This suggests partial decomposition of **SnS**_**2**_ **/DMA** due to the release of the organic content (dimethylamine) at temperatures above 100 °C. The results from VT-PXRD measurements align with those obtained from the thermal analysis discussed below.

Thermogravimetric analysis (TGA) was performed to determine H_2_O and dimethylamine content in the new materials. The TGA data for **SnS**_**2**_ **/DMA** (Figure S4) indicate two stages of weight loss, with the first weight loss from 35 to 102 °C ascribed to the removal of H_2_O (∼2.6%) and the following weight loss (∼14.6%) attributed to the release of dimethylamine. Based on this data, the dimethylamine and water contents of the material were calculated at 0.7 and 0.3 mol per formula unit (see Supporting Information). As for **SnS**_**2**_ **/acid**, the material shows only one weight loss stage from 35 to 106 °C (Figure S5) assigned to removing H_2_O (∼6.3%). The absence of weight loss at higher temperatures indicates that **SnS**_**2**_ **/acid** does not contain dimethylamine.

**SnS**_**2**_ **/DMA** has an almost neutral surface charge (showing a zeta potential slightly above zero, namely +0.353 mV at pH ∼ 7, Figure S6), whereas **SnS**_**2**_ **/acid** exhibits a negative surface charge with a zeta potential of -30.4 mV at pH ∼ 7 (Figure S7). The origin of the negative surface charge of **SnS**_**2**_ **/acid** is likely the Sn atoms’ leaching from the surface of **SnS**_**2**_ **/DMA** upon its treatment with the concentrated acidic solution. Energy-dispersive X-ray Spectroscopy (EDS) and X-ray Fluorescence Spectroscopy (XRF) (Figure S8) data revealed a Sn/S ratio of ∼ 1/2 for **SnS**_**2**_ **/acid** (similar results were also obtained for **SnS**_**2**_ **/DMA**). Thus, the Sn deficiency is likely too small to be determined considering the accuracy of the EDS and XRF measurements (5–10%).^46^

The IR spectra of **SnS**_**2**_ **/DMA** and **SnS**_**2**_ **/acid** are given in Figure S9. **SnS**_**2**_ **/DMA** has vibration bands at 3400, 2900, and 1400 cm^–1^, which can be attributed to −N–H, −C–H, and −C–N bonds, respectively. These vibration bands are significantly weaker in **SnS**_**2**_ **/acid**’s spectrum, confirming the almost quantitative removal of dimethylamine from **SnS**_**2**_ **/DMA**.

Field Emission-Scanning Electron Microscopy (FE-SEM) demonstrates a typical layer morphology for **SnS**_**2**_ **/DMA** and **SnS**_**2**_ **/acid**. In particular, the particles have a sheet-like morphology and are stacked on each other (Figures S10 and S11). **SnS**_**2**_ **/DMA** has an average particle size of about 80 μm, whereas **SnS**_**2**_ **/acid** displays significantly smaller particles of around 7–7.5 μm. This difference in particle size can be attributed to the stirring process, which causes exfoliation of **SnS**_**2**_ **/DMA** and leads to the formation of smaller fragments in **SnS**_**2**_ **/acid**. Energy-dispersive X-ray Spectroscopy (EDS) data (Figures S12 and S13) revealed the presence of Sn, S, and N atoms in **SnS**_**2**_ **/DMA** and Sn and S atoms in **SnS**_**2**_ **/acid**.

X-ray Photoelectron Spectroscopy (XPS) measurements were performed for pristine **SnS**_**2**_ (synthesized via a solid-state reaction), **SnS**_**2**_ **/DMA,** and **SnS**_**2**_ **/acid**. The S spectrum of the nonintercalated **SnS**_**2**_ indicates two peaks, at 162.8 and 161.6 eV, assigned to S 2p_1/2_ and S 2p_3/2_ core-level signals (Figure S14A). The characteristic Sn^4+^ peaks, Sn 3d_3/2_ and Sn 3d_5/2_ core-level signals, are presented in Figure S14B with binding energies of 495.1 and 486.7 eV, respectively. The S spectrum (Figure S15A) for **SnS**_**2**_ **/DMA** consists of two peaks, at 161.9 and 160.7 eV, attributed to S 2p_1/2_ and S 2p_3/2_ core-level signals. These values differ around 1 eV from those of nonintercalated SnS_2_, likely because of possible interactions of the intralayer S^2–^ ligands with the intercalated dimethylamine and water molecules. The binding energies of the characteristic Sn^4+^ peaks, Sn 3d_3/2_ and 3d_5/2_, are 494.4 and 486.0 eV, respectively (Figure S15B), negatively shifted compared to those for the nonintercalated material. This shift can also be attributed to the effect of the intercalated amine/water molecules. The presence of Sn atoms in other oxidation states besides (IV) is excluded from the Mössbauer data discussed below. In the N 1s spectrum (Figure S15C), the main peak centers at 401.2 eV, positively shifted compared to the expected value for neutral amines. This shift can be ascribed to the formation of relatively strong hydrogen bonds between dimethylamine molecules or dimethylamine and H_2_O molecules present in the interlayer spacing of **SnS**_**2**_ **/DMA**. The existence of protonated dimethylamine is not likely because **SnS**_**2**_ **/DMA** has a neutral surface charge, indicating neutral metal sulfide layers and intercalated amines. As for **SnS**_**2**_ **/acid**, the 162.5 and 161.1 eV peaks can be attributed to S 2p_1/2_ and S 2p_3/2_ core-level signals, respectively (Figure S15D). We thus observe much less shift for the sulfur signals of **SnS**_**2**_ **/acid** vs those of **SnS**_**2**_ **/DMA**, which reflects the absence of intercalated dimethylamine molecules in the first material. The binding energies of the Sn 3d_3/2_ and Sn 3d_5/2_ core-level signals are 494.9 and 486.5 eV, like those observed for the nonintercalated SnS_2_ (Figure S15E). In addition, XPS confirmed the successful removal of dimethylamine from **SnS**_**2**_ **/DMA** (Figure S16B), as nitrogen is hardly seen in the XPS spectrum of **SnS**_**2**_ **/acid**.

^119^Sn Mössbauer spectra of the pristine **SnS**_**2**_, **SnS**_**2**_ **/DMA,** and **SnS**_**2**_ **/acid** samples recorded at 80 K are shown in Figure 3. The spectrum of the pristine **SnS**_**2**_ sample, which was synthesized following the solid-state reaction path to be used as the standard, was fitted with one component having fixed zero quadrupole splitting (QS), as expected by the nature of the high symmetric regular SnS_6_ octahedra found in the SnS_2_ structure.^47–49^ Table S1 gives the resulting Mössbauer parameters from the fittings of all spectra. The isomer shift (IS) value for the singlet is 1.06(2) mm s^–1^, which falls within the expected values for the Sn^4+^ ions in this phase.^47–49^ However, a broadening of the resonant lines is observed for the spectra of the **SnS**_**2**_ **/DMA** and **SnS**_**2**_ **/acid** samples compared to that of the pristine **SnS**_**2**_ sample. Consequently, a set of two components, composed of a singlet and a doublet, fit these spectra adequately. From Table S1, the resulting Mössbauer parameters reveal that both components correspond to Sn^4+^ ions. For the singlet, the IS values are only slightly shifted relative to that found for the pristine **SnS**_**2**_ sample, but for the IS of the doublet, the shift is higher for both samples. From the nature of these components, relative to the properties of the corresponding samples, we can assign the singlet to those Sn^4+^ ions situated at the regular octahedra of the SnS_2_ layered structure. At the same time, the appearance of the nonvanishing QS values for the doublet is attributed to the distortions induced by some of the SnS_6_ octahedra because of the presence of the interlayer DMA and H_2_O molecules and the defects that should be unavoidably created by the subsequent insertion and/or extraction of these molecules at the interlayer space. These defects should mainly include Sn^4+^ and/or S^2–^ ion vacancies. From the values of the absorption areas of these two components, resulting in the **SnS**_**2**_ **/DMA** and **SnS**_**2**_ **/acid** spectra, it can be suggested that the population of the Sn^4+^ ions that are affected and unaffected, respectively, by the insertion and extraction of the DMA and H_2_O molecules is close to 1:1; that is, about half of the Sn^4+^ ions are strongly affected by this procedure.

**Figure 3:**
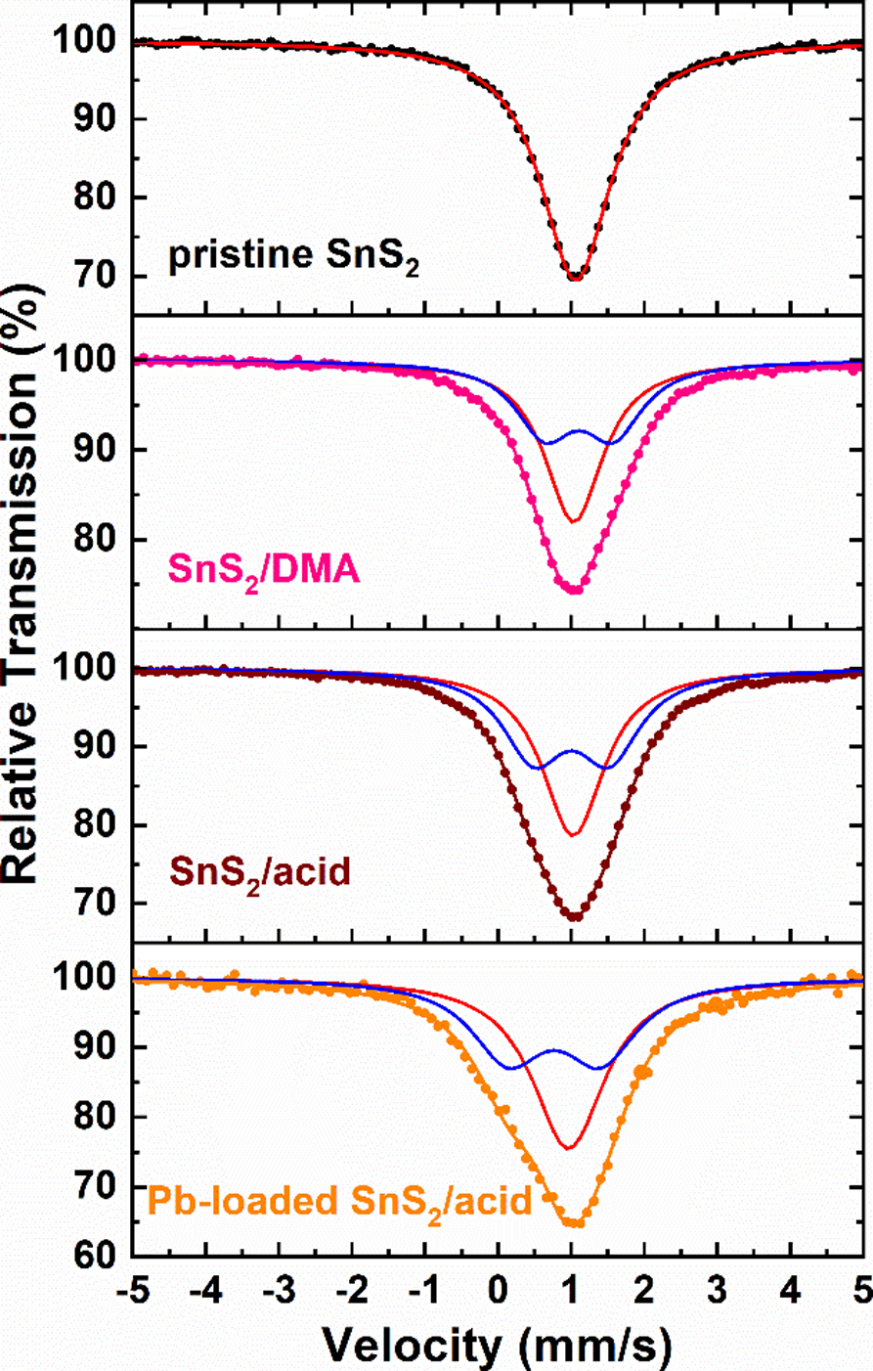
^119^Sn Mössbauer spectra of the pristine **SnS**_**2**_, **SnS**_**2**_ **/DMA**, **SnS**_**2**_ **/acid**, and **Pb-loaded SnS**_**2**_ **/acid** samples recorded at 80 K. The points represent the experimental data, while the colored continuous lines correspond to the components that fit the spectra, as the text describes.

### 3.2 Metal Sulfide-Cotton Fabric Composites

**SnS**_**2**_ **/DMA** and **SnS**_**2**_ **/acid** were immobilized onto cotton fabric textiles with poly­(methyl methacrylate) (PMMA), an inexpensive and nontoxic adhesive agent.^50^ The PXRD patterns (Figure 4A,B) reveal the successful immobilization of **SnS**_**2**_ **/DMA** and **SnS**_**2**_ **/acid** onto the cotton substrate. In addition, FE-SEM images of **SnS**_**2**_ **/DMA PMMA@Cotton Fabric** (Figure S17) and **SnS**_**2**_ **/acid PMMA@Cotton Fabric** (Figure S19) revealed that the cotton’s surface is extensively coated with MS sheet-like particles, as further validated by EDS analysis (Figures S18 and S20).

**Figure 4:**
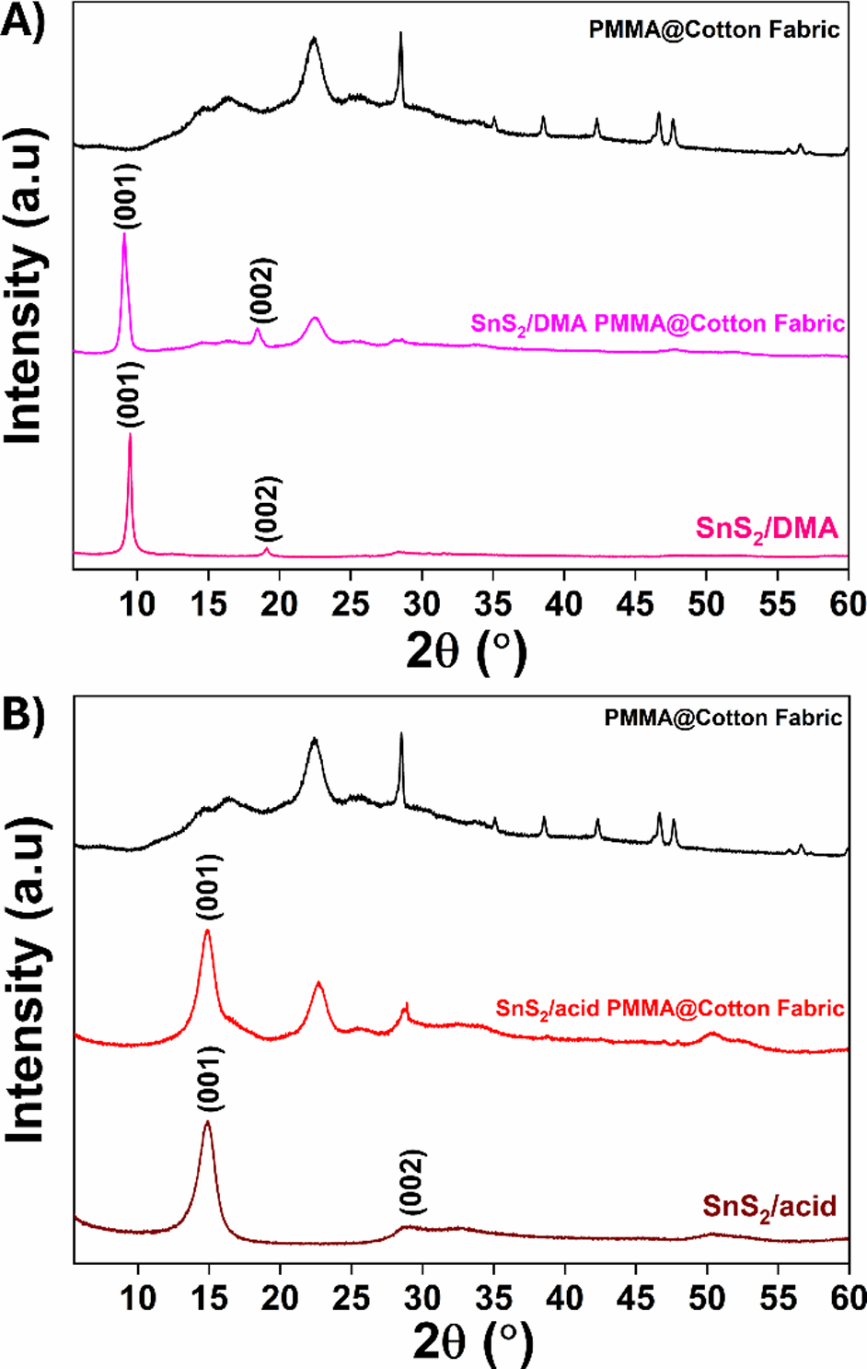
PXRD pattern of (A) **SnS**_**2**_ **/DMA** (pink), **SnS**_**2**_ **/DMA PMMA@Cotton fabric** (light pink), PMMA@Cotton fabric (black), and (B) **SnS**_**2**_ **/acid** (wine), **SnS**_**2**_ **/acid PMMA@Cotton fabric** (red), and PMMA@Cotton fabric (black).

### 3.3 Batch Sorption Studies

Metal sulfides containing abundant sulfur sites are ideal sorbents for soft or relatively soft species such as Pb^2+^ ions. Thus, **SnS**_**2**_ **/DMA** and **SnS**_**2**_ **/acid** were evaluated in detail for their Pb^2+^ sorption properties. Further motivation for studying **SnS**_**2**_ **/acid** as a cation sorbent was its significant negative surface charge, particularly useful for cation sorption. Similar studies were also conducted for the pristine, nonintercalated **SnS**_**2**_ material for comparison.

### 3.4 Kinetic Studies

The results of the kinetics sorption study for **SnS**_**2**_ **/DMA** with a low initial Pb^2+^ concentration (1 ppm) revealed fast capture of Pb^2+,^ with the equilibrium reached within the first 4 min of contact (Figure 5A). Interestingly, **SnS**_**2**_ **/DMA** removes 97.6% of the initial concentration of Pb^2+^. The kinetics for the sorption of Pb^2+^ was fitted with Lagergren’s first-order model (eq 1, SI, *k* = 1.53 s^–1^). Using higher Pb^2+^ concentrations (81.8 ppm), the sorption kinetics was slower. Nevertheless, **SnS**_**2**_ **/DMA** could efficiently capture 99.7% of the initial Pb^2+^ concentration (Figure S21) within 480 min (8 h) of solid/solution contact, but the data could not be fitted to any kinetics model. **SnS**_**2**_ **/acid** demonstrated outstanding speed and efficiency as a Pb^2+^ sorbent, achieving a 99.9% removal rate at 1 ppm of Pb^2+^, with equilibrium reached within 30 s of contact (Figure 6A). Hence, the results could not be fitted to any kinetics model due to the extremely fast sorption of Pb^2+^ by **SnS**_**2**_ **/acid**. At higher concentrations (81.8 ppm), the equilibrium was attained within 480 min of contact, achieving 99.7% removal of the initial Pb^2+^ amount (Figure S22). Lagergren’s first-order model fits the kinetic data (eq 1, SI, *k* = 0.007 s^–1^).

**Figure 5:**
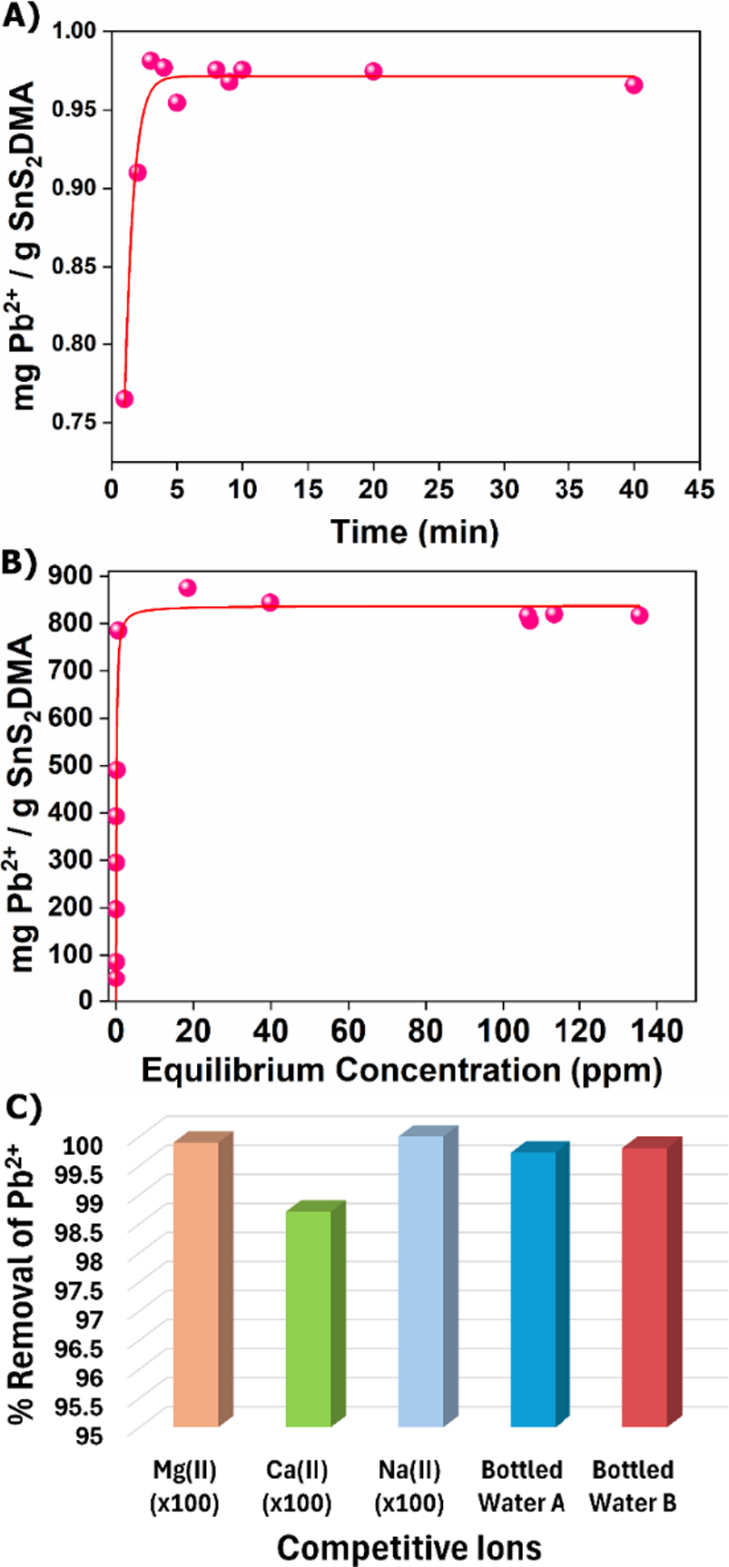
(A) Kinetics of Pb^2+^ sorption for **SnS**_**2**_ **/DMA** (*C*_initial_ of Pb^2+^= 1 ppm, pH ∼ 7), (B) Isotherm Pb^2+^ sorption data for **SnS**_**2**_ **/DMA**. The red line signifies the data fitted with the Langmuir model (*R* ^2^ = 0.73, *q*_e_ = 838.0 ± 67.0 mg g^–1^ and *b* = 10.1 ± 8.2 L mg^–1^ (contact time, *t* = 24 h), (C) Pb^2+^ sorption data for **SnS**_**2**_ **/DMA** in the coexistence of various cations (100-fold excess) and for artificially contaminated bottled water samples (*C*_initial_ of Pb^2+^ = 1 ppm, pH ∼ 7), Composition of bottled water A: Ca^2+^ = 34.6 ppm, Mg^2+^ = 1.98 ppm, Na^+^= 1.53 ppm, K^+^= 0.18 ppm, and of bottled water B: Ca^2+^ = 80.7 ppm, Mg^2+^ = 5.34 ppm, Na^+^ = 2.24 ppm, K^+^ = 0.6 ppm.

**Figure 6:**
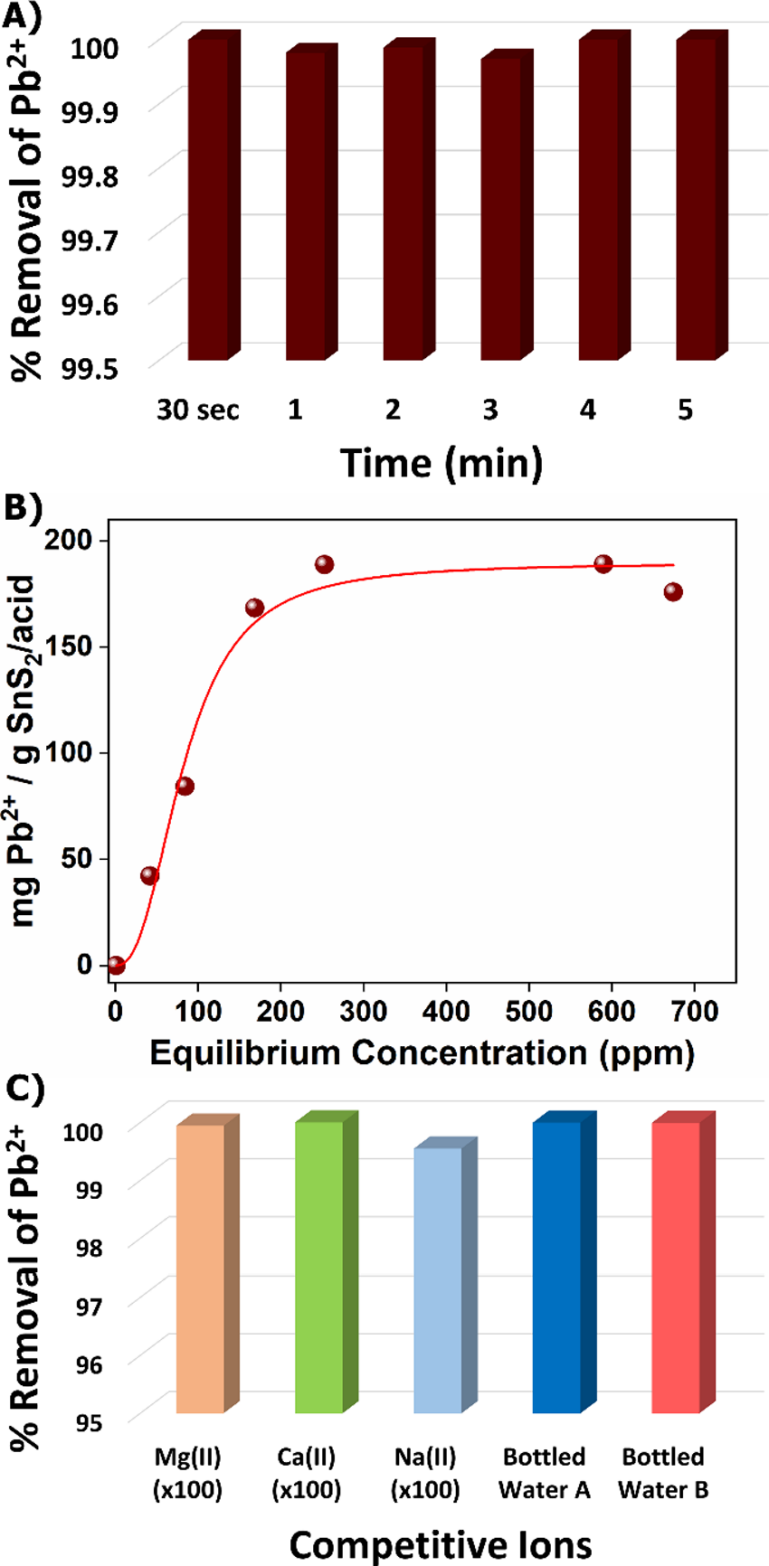
(A) Kinetics of Pb^2+^ sorption for **SnS**_**2**_ **/acid** (*C*_initial_ of Pb^2+^= 1 ppm, pH ∼ 7), (B) Isotherm Pb^2+^ sorption data for **SnS**_**2**_ **/acid**. The red line signifies the data fitted with the Langmuir–Freundlich model (*R* ^2^ = 0.97, *q*_e_ = 190.0 ± 9.0 mg g^–1^, *b* = 0.01 ± 0.001 L mg^–1^ and *n* = 0.40 ± 0.09 (contact time, *t* = 24 h), (C) Pb^2+^ sorption data for **SnS**_**2**_ **/acid** in the coexistence of various cations (100-fold excess) and for artificially contaminated bottled water samples (*C*_initial_ of Pb^2+^= 1 ppm, pH ∼ 7). The composition of bottled water samples is provided in the caption of Figure 5.

The pristine **SnS**_**2**_ was also tested for its Pb^2+^ sorption properties. At a low Pb^2+^ concentration (1 ppm), the material could capture only 37.3% of the initial concentration in 60 min. At higher concentrations (81.8 ppm), the Pb^2+^ sorption can be described with Lagergren’s first-order equation (eq 1, SI, *k* = 0.003 s^–1^). The equilibrium is achieved after 24 h of contact with 99.9% removal of Pb^2+^ (Figure S23). Comparing the kinetic constants of **SnS**_**2**_ **/acid** (*k* = 0.007 s^–1^) and the nonintercalated **SnS**_**2**_ (*k* = 0.003 s^–1^), it is evident that **SnS**_**2**_ **/acid** reacts around twice as fast as **SnS**_**2**_ under the given conditions. This can be justified since **SnS**_**2**_ **/acid** has a negative surface charge that facilitates the rapid attraction of cationic species.

### 3.5 Sorption Isotherm Studies

The Pb^2+^ sorption isotherm data for **SnS**_**2**_ **/DMA** were well fitted to the Langmuir isotherm model (eq 2, SI), exhibiting a maximum sorption capacity calculated to be 838.0 ± 67.0 mg of Pb^2+^ per gram of the material (Figure 5B). The sorption isotherm data of **SnS**_**2**_ **/acid** can be fitted with the Langmuir–Freundlich model (eq 3, SI) (Figure 6B). The results indicate a maximum sorption capacity of 190.0 ± 9.0 mg Pb^2+^ per gram of **SnS**_**2**_ **/acid**. A sorption isotherm study was also conducted for pristine **SnS**_**2**_. The Langmuir isotherm model (eq 2, SI) was used to fit the Pb^2+^ sorption data for SnS_2_ (Figure S24), revealing a maximum sorption capacity of 250.0 ± 32.0 mg Pb^2+^ per gram of **SnS**_**2**_.

### 3.6 Selectivity Studies and Application in Real Water Samples

Another factor that plays a vital role in the efficiency of a sorbent toward toxic metal ions is the coexistence of various cations such as Mg^2+^, Ca^2+^, and Na^+^. Therefore, we also investigated the sorption capability of **SnS**_**2**_ **/DMA** and **SnS**_**2**_ **/acid** in the presence of the above competitive cations. **SnS**_**2**_ **/DMA** retains its sorption capability even in a 100-fold excess of Na^+^, Mg^2+^, and Ca^2+^ (removal percentages of 100, 98.4, and 98.7%, respectively) (Figure 5C). For **SnS**_**2**_ **/acid**, the Pb^2+^ sorption is not affected by the coexistence of Ca^2+^, Na^+^ and Mg^2+^, as the removal percentages remained exceptionally high (99.5–100%) (Figure 6C) in the presence of these competitive cations. As the final step in this study, we conducted sorption experiments using bottled water samples (A and B) spiked with Pb^2+^ ions to simulate wastewater; such samples contain a variety of competitive cations like Ca^2+^, Na^+^, and Mg^2+^, in significant excess (Sample A: 34.6, 2.0, 1.5 -fold, Sample B: 80.7, 5.3, 2.2-fold, respectively) compared to Pb^2+^, as well as several anions such as Cl^–^, NO_3_^–^, HCO_3_^–^, and SO_4_^2–^. Remarkably, despite the presence of these competitive ions, both **SnS**_**2**_ **/DMA** (Figure 5C) and **SnS**_**2**_ **/acid** (Figure 6C) can efficiently remove Pb^2+^ exhibiting high removal percentages (99.7 and 99.8% for **SnS**_**2**_ **/DMA**, 99.9 and 99.9% for **SnS**_**2**_ **/acid**, for bottled water A and B respectively).

### 3.7 Variable pH Sorption Studies

We also studied the effect of pH on Pb^2+^ sorption by the new metal sulfide materials. The results indicated that **SnS**_**2**_ **/DMA** could efficiently capture Pb^2+^ from pH 4 to 7, indicating a removal percentage of 99.9%, whereas at pH = 3, the removal percentage was reduced to 96.3% (Figure S25). According to the results depicted in Figure S26, the Pb^2+^ removal percentages by **SnS**_**2**_ **/acid** were 81.4–99.4% at pH 4 to 7. In contrast, at pH = 3, the removal percentage (12.9%) significantly decreased.

Finally, we should note that no leaching of dimethylamine was detected in Pb^2+^ solutions treated by **SnS**_**2**_ **/DMA**, as determined with ^1^H NMR Spectroscopy (Figure S27).

### 3.8 Sorption under Continuous Flow conditions

As mentioned above, metal sulfides in powder form are unsuitable for wastewater treatment applications. Thus, the cotton composites of **SnS**_**2**_ **/DMA** and **SnS**_**2**_ **/acid** were prepared. At first, **SnS**_**2**_ **/DMA-PMMA@Cotton fabric** and **SnS**_**2**_ **/acid-PMMA@Cotton fabric** were tested for their Pb^2+^ removal properties under batch conditions using bottled water samples intentionally spiked with 10 ppm of Pb^2+^. The results showed that within only 1 h of contact, the composites could successfully remove Pb^2+^ from the contaminated bottled water samples (removal percentage of >98%). In contrast, the PMMA@Cotton fabric (containing no metal sulfide) showed no Pb^2+^ sorption. Encouraged by the promising results, we investigated the cotton composites’ performance for decontaminating bottled water samples spiked with Pb^2+^ (initial Pb^2+^ concentration range: 8.5–11.2 ppm) under continuous flow conditions. Thus, we set up a column with a stationary phase consisting of six MS-cotton samples and 29.4 g of sea sand (Figure 7A).

**Figure 7:**
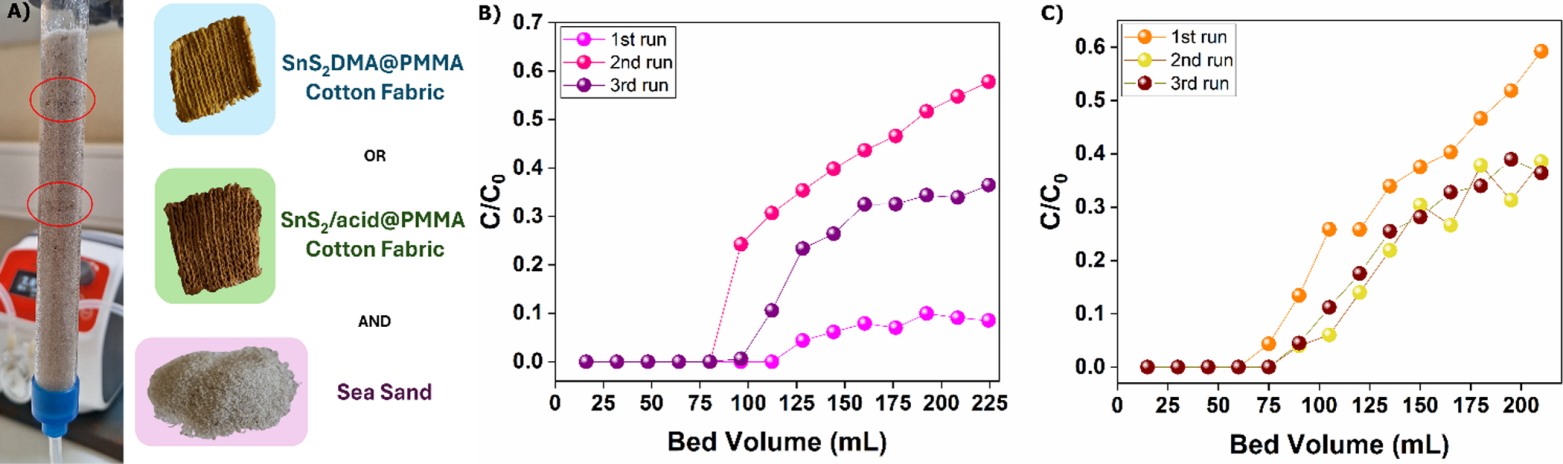
(A) Experimental setup used for Pb^2+^ sorption studies under continuous flow conditions (6 pieces of **SnS**_**2**_ **/DMA PMMA@Cotton Fabric** and **SnS**_**2**_ **/acid PMMA@Cotton Fabric** and 29.4 g of sea sand were used in each column). Breakthrough curves for three column runs of (B) **SnS**_**2**_ **/DMA-PMMA@Cotton Fabric** *(C*_initial_ of Pb^2+^ of the 1st, 2nd and 3rd run, respectively: 10.7, 11.0, and 10.2 ppm) and (C) **SnS**_**2**_ **/acid-PMMA@Cotton Fabric** (*C*_initial_ of Pb^2+^ of the 1st, 2nd and 3rd run respectively: 8.5, 9.0, and 10.0 ppm) (pH ∼ 5.5, flow rate = 0.8 mL/min, one-bed volume = 16 and 15 mL respectively). The lines are included for visual guidance.

Passing ∼112 mL of the wastewater simulant solution through the **SnS**_**2**_ **/DMA-PMMA@Cotton Fabric** column, no Pb was detected in the effluent (Figure 7B), and the breakthrough capacity (eq 5, SI) was found to be 72.3 mg g^–1^. The column was regenerated with 1 M HCl acid solution. Then, a second run was performed, indicating no Pb in the effluent after passing ∼80 mL of the solution, and the breakthrough capacity was determined to be 53.0 mg g^–1^. The column largely retained its sorption capacity even for a third run, showing a breakthrough capacity of 49.4 mg g^–1^.

Regarding the **SnS**_**2**_ **/acid PMMA@Cotton Fabric** column (Figure 7C), the breakthrough capacities for three successive runs were determined to be 29.8, 39.2, and 49.7 mg g^–1^ for the first, second, and third column run, respectively. These unexpected sorption properties for the **SnS**_**2**_ **/acid PMMA@Cotton Fabric** column, indicating a slight increase in the breakthrough capacity in the second and third runs, are reproducible. This abnormal behavior of the column may be tentatively attributed to the rise of Sn deficiency and the negative surface charge (favoring the interaction of the metal sulfide with the Pb^2+^ cations) of the sorbent upon the regeneration process involving washing with a highly acidic solution (HCl 1M). Overall, the above column sorption results indicate the reusability of the new metal sulfide sorbents, in contrast to previous works revealing that metal sulfides cannot be regenerated and reused for Pb^2+^ sorption.^36,39,51^

### 3.9 Mechanism of Pb^2+^ Sorption: Characterization of the Pb-Loaded MSs

The successful binding of Pb^2+^ ions on the MSs and their cotton-fabric composites was confirmed by PXRD, XPS, EDS, and ^119^Sn Mossbauer Spectroscopy. EDS analysis revealed Pb^2+^ ions in the Pb-loaded MSs (Figures S28 and S29). The PXRD data showed that at 10 ppm of Pb^2+^, the structure of **SnS**_**2**_ **/DMA** remains intact, whereas, using 50 ppm of Pb^2+^ or above, a partial decomposition of the material and the formation of the PbS phase were observed (Figure 8A). As for **SnS**_**2**_ **/acid,** no structural alterations were noted when the material was treated with up to 50 ppm of Pb^2+^. However, the treatment of **SnS**_**2**_ **/acid** with a Pb^2+^ solution of 100 ppm resulted in a partial decomposition of the material to PbS (Figure 8B). Similar results were obtained for the Pb-loaded MS-PMMA@Cotton Fabric composites, as revealed by their PXRD data (Figure 8C,D). The XPS data for **Pb-loaded SnS**_**2**_ **/acid** and **Pb-loaded SnS**_**2**_ **/DMA** confirmed the presence of Pb 4f_5/2_ and Pb 4f_7/2_ core-level signals (Figure S30C,G), with binding energies of 142.7 and 137.8 eV, and 142.2 and 137.5 eV, respectively. Moreover, the binding energies for the S 2p_1/2_ and 2p_3/2_ core-level signals of **Pb-loaded SnS**_**2**_ **/acid** (Figure S30A) were found to be 162.5 and 161.3 eV, respectively, with the latter value positively shifted ∼0.2 eV compared to the lead-free material (Figure 9B). Similarly, for **Pb-loaded SnS**_**2**_ **/DMA**, the S spectrum (Figure S30D) consists of two peaks, at 162.4 and 161.2 eV, attributed to S 2p_1/2_ and S 2p_3/2_ core-level signals. Both binding energies exhibited ∼0.5 eV positive shift compared to the corresponding values for **SnS**_**2**_ **/DMA**. The shift to higher binding energies in both Pb^2+^-loaded materials may be attributed to the strong interactions of the sorbed Pb^2+^ with the S^2–^ ligands, involving an electron transfer from the sulfide ligands to the Pb^2+^ cations (Figure 9A).

**Figure 8:**
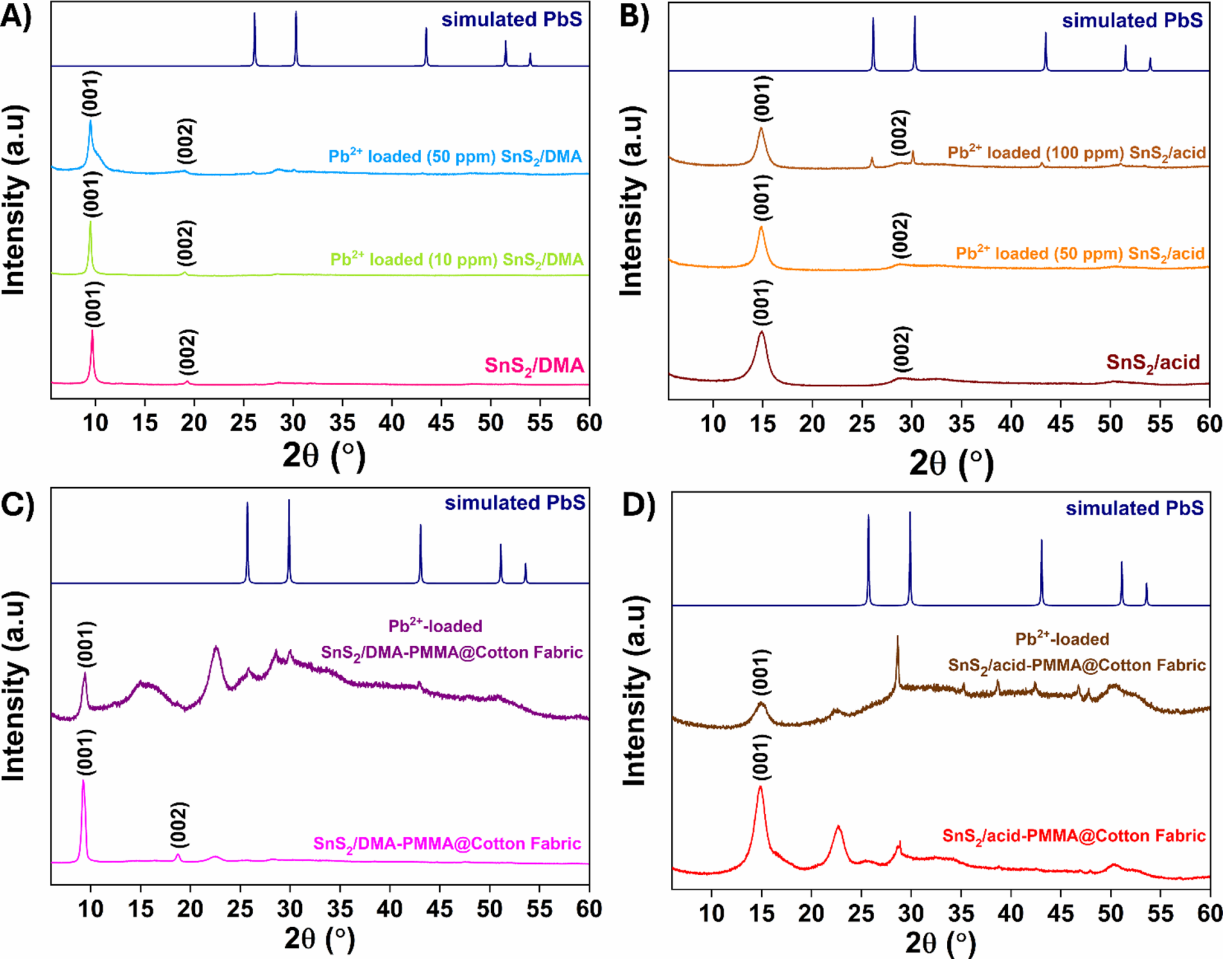
PXRD patterns of (A) **Pb-loaded SnS**_**2**_ **/DMA**, (B) **Pb-loaded SnS**_**2**_ **/acid**, (C) **Pb-loaded SnS**_**2**_ **/DMA PMMA@Cotton Fabric** (*C*_initial_ of Pb^2+^= 10 ppm), (D) **Pb-loaded SnS**_**2**_ **/acid PMMA@Cotton Fabric** (*C*_initial_ of Pb^2+^= 10 ppm) and simulated PbS.

**Figure 9:**
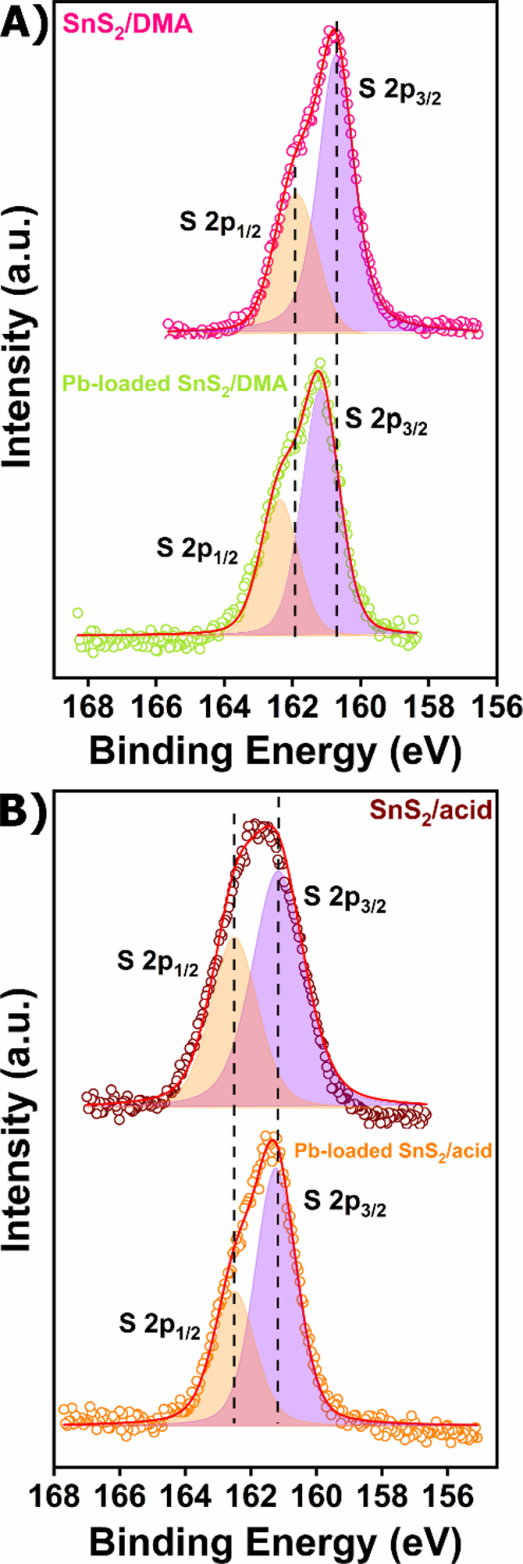
Comparison of the XPS spectra of (A) S 2p_1/2_ and 2p_3/2_ of **SnS**_**2**_ **/DMA** and **Pb-loaded SnS**_**2**_ **/DMA** and (B) S 2p_1/2_ and 2p_3/2_ of **SnS**_**2**_ **/acid** and **Pb-loaded SnS**_**2**_ **/acid**.

Moreover, the Mössbauer spectrum of the **Pb-loaded SnS**_**2**_ **/acid** sample appearing in Figure 3 acquires a higher broadening of its resonant lines than the **SnS**_**2**_ **/DMA** and **SnS**_**2**_ **/acid** samples spectra. We used the same model composed of a singlet and a doublet for the later samples to fit this spectrum. The resulting Mössbauer parameters listed in Table S1 reveal more significant shifts of the IS values for both components and a further increase of the QS value for the doublet compared to those found for the **SnS**_**2**_ **/DMA** and **SnS**_**2**_ **/acid** samples. This implies that the sorbed Pb^2+^ ions in the **Pb-loaded SnS**_**2**_ **/acid** sample should affect even more drastically the properties of the Sn^4+^ ions through their connection to the S^2–^ ligands, as proposed following the XPS analyses above. This is reflected in the electronic configuration of the Sn^4+^ ions through the higher shift in their IS values and the further increase of distortions to their local environment through the higher QS values observed for the doublet. Therefore, the remarkable Pb^2+^ capture by **SnS**_**2**_ **/DMA** results from the strong interactions between the soft S^2–^ ligands with the Pb^2+^ ions. For **SnS**_**2**_ **/acid**, the strong affinity of S^2–^ for Pb^2+^ ions and the negative surface charge, which induces electrostatic interactions with the Pb^2+^ ions, facilitate the Pb^2+^ sorption. However, with a higher concentration of Pb^2+^ ions in both cases, the formation of the PbS phase also contributes to the capture of Pb^2+^ from the contaminated samples.^52^

Finally, an explanation should be provided for the reusability of the materials reported in this work, in contrast to known metal sulfides that cannot be reused for Pb^2+^ sorption. Most known sorbents are based on anionic metal sulfide layers, and the removal mechanism of Pb^2+^ ions is mainly attributed to ion-exchange with the intercalated cations. Strong Pb–S bonds within the interlayer spacing of these materials make regeneration not feasible under mild conditions, whereas using more intense conditions (e.g., concentrated acidic solutions) results in the decomposition of the materials.^18–20^ In contrast, the compounds presented in this work do not sorb Pb^2+^ via ion-exchange but through Pb^2+^ interactions with the S atoms in the surface of the materials. As Pb^2+^ ions are located on the surface rather than the interlayer space of the metal sulfide, their desorption can be performed under relatively mild conditions, thus avoiding the deterioration of the metal sulfide structure. As a result, the materials can be easily regenerated, without structure deterioration, and reused.

### 3.10 Comparison of the New SnS_2_-Based Materials with Other Pb^2+^ Sorbents

At this point, comparing the Pb^2+^ sorption properties of the new SnS_2_-based materials with those of other sorbents would be helpful. Tables S2 and S3 present the most important properties (sorption capacities, equilibrium time, selectivity, reusability, mass of the sorbent, flow rate, and initial concentration) of various materials investigated for their Pb^2+^ removal efficiency under batch and flow conditions, respectively. **SnS**_**2**_ **/DMA** exhibits a high sorption capacity of 838.0 mg g^–1^, one of the highest among the most effective Pb^2+^ sorbents.^36,53,54^ Some reported sorbents may achieve higher sorption capacities but are not reusable. In contrast, **SnS**_**2**_ **/DMA** in the form of its composite with cotton fabric can be regenerated and reused for Pb^2+^ sorption under flow conditions. Although **SnS**_**2**_ **/DMA** requires longer equilibrium times at high Pb^2+^ concentrations (8 h), it achieves exceptionally rapid equilibrium at low concentrations of Pb^2+^ (4 min for concentrations <1 ppm). In addition, it exhibits high selectivity for Pb^2+^ ions, among various coexisting ions such as Ca^2+^, Na^+^ and Mg^2+^.

On the other hand, **SnS**_**2**_ **/acid** displays a lower sorption capacity of 190.0 mg g^–1^ compared to the Pb^2+^ sorbents presented in Table S2. However, it achieves equilibrium times as fast as 30 s at low concentrations of Pb^2+^ (1 ppm). In addition, it exhibits exceptional selectivity toward Pb^2+^ ions, with a range of coexisting cations like Ca^2+^, Na^+^, and Mg^2+^ in a high excess. Lastly, **SnS**_**2**_ **/acid** can be immobilized on cotton fabrics and used effectively to remove Pb^2+^ ions under continuous flow conditions several times.

## 4 Conclusions

In conclusion, we successfully synthesized new SnS_2_-based materials, **SnS**_**2**_ **/DMA**, through a one-step solvothermal reaction and **SnS**_**2**_ **/acid** by treating **SnS**_**2**_ **/DMA** with acid. These materials displayed fast sorption kinetics (≤4 min) and high sorption capacities (838.0 and 190.0 mg g^–1^). Additionally, they exhibited exceptional selectivity for Pb^2+^ amidst various coexisting ions, suggesting their potential for practical applications. The remarkable affinity of these new materials for Pb^2+^ ions is mainly due to the strong interactions of the soft S^2–^ ligands with the Pb^2+^ ions. Furthermore, in the case of **SnS**_**2**_ **/acid**, its negatively charged surface further enhances this affinity by inducing electrostatic interactions with the Pb^2+^ ions. Aiming at applications in wastewater treatment, we immobilized the new metal sulfides onto cotton fabric substrates using PMMA as an adhesive agent. This immobilization process was applied for the first time for metal sulfide materials. The composite materials were employed as a stationary phase (along with sea sand) in sorption columns and proved highly effective in remediating bottled water samples artificially contaminated with Pb^2+^ ions under continuous flow conditions. Notably, the metal sulfide-based materials could be regenerated and reused for Pb^2+^ sorption, representing a significant breakthrough for this family of sorbents, as this property was not reported before the present work. These findings encourage us to continue working on metal sulfide-cotton composites, which seem promising as filters for rapidly removing heavy metals from aqueous media. Unlike conventional powdered sorbents, these composite materials are more suitable for practical wastewater treatment applications. Future work will focus on developing new composite materials and evaluating their performance in real wastewater conditions, particularly regarding reusability and scalability.

## Supplementary Material



## Notes

Additional experimental details, materials, and methods, PXRD patterns, TGA and zeta potential diagrams, FE-SEM images, ^1^H NMR, EDS, IR, and XPS spectra, ^119^Sn Mössbauer parameters and XRF measurements tables, kinetics, isotherm, pH sorption data, and tables with comparisons of Pb^2+^ sorbents (PDF)
