# Supplementary material for: Reusable SnS2‑Based Cotton Fabric Composites for Efficient Decontamination of Water from Lead Ions under Continuous Flow Conditions
Source: Langmuir. 2025 May 28;41(22):14449–59. doi: 10.1021/acs.langmuir.5c01540 (PMC12164348; doi:10.1021/acs.langmuir.5c01540)
Supplement: Supplementary file 1 [file la5c01540_si_001.pdf]

Supporting Information

Reusable SnS_2-based cotton fabric composites for efficient decontamination of water from lead ions under continuous flow conditions

Vasiliki I. Karagianni[a], Efthymia Toti[a], Christos Dimitriou[b], Yiannis Deligiannakis[b], Alexios P. Douvalis[b] and Manolis J. Manos[a]*

a. Department of Chemistry, University of Ioannina, Ioannina, GR-45110, Greece

b. Department of Physics, University of Ioannina, Ioannina, GR-45110, Greece

Corresponding author e-mail: emanos@uoi.gr

Number of pages: 28
Number of figures: 30
Number of schemes: 0
Number of tables: 3

Table of Contents

Experimental Section S4

Material Characterization S4

Methods S5

Calculation of the band gap energy S5

Isolation of the Pb-loaded materials S5

Batch sorption studies S5

Kinetic studies S5

Isotherm study S6

Competitive sorption and pH studies S6

Sorption under continuous flow conditions S6

Figure S1. PXRD patterns of **SnS_2** (black), **SnS_2/DMA** (pink) and **SnS_2/acid** (wine). S7

Figure S2. VT-PXRD patterns of **SnS_2/DMA** at room temperature (RT), 50 and 100℃. S8

Figure S3. VT-PXRD patterns of **SnS_2/DMA** at room temperature (RT), 150-300 ℃ and the simulated pattern of SnS_2. .... S8

Figure S4. TGA data of **SnS_2/DMA** measured in N_2 atmosphere. S9

Figure S5. TGA data of **SnS_2/acid** measured in N_2 atmosphere. S10

Figure S6. Zeta-potential diagram of **SnS_2/DMA**. S11

Figure S7. Zeta-potential diagram of **SnS_2/acid**. S11

Figure S8. XRF measurements of **SnS_2/acid**. S11

Figure S9. IR-spectra of **SnS_2/DMA** (pink) and **SnS_2/acid** (wine). The peaks around 2000 cm^{-1} are attributed to the diamond ATR.[8] S12

Figure S10. FE-SEM images of **SnS_2/DMA**. S12

Figure S11. FE-SEM images of **SnS_2/acid**. S12

Figure S12. EDS spectrum of **SnS_2/DMA**. S13

Figure S13. EDS spectrum of **SnS_2/acid**. S13

Figure S14. High-resolution XPS spectra of a) S $2p_{1/2}$ and $2p_{3/2}$ and b) Sn $3d_{3/2}$ and $3d_{5/2}$ of pristine **SnS_2**. S13

Figure S15. High resolution XPS spectra of a) S $2p_{1/2}$ and $2p_{3/2}$ b) Sn $3d_{3/2}$ and $3d_{5/2}$ and c) N 1s of **SnS_2/DMA**, d) S $2p_{1/2}$ and $2p_{3/2}$ and e) Sn $3d_{3/2}$ and $3d_{5/2}$ of **SnS_2/acid**. S14

Figure S16. a) XPS survey of **SnS_2/DMA**, **SnS_2/acid**, **Pb-loaded SnS_2/acid** and **Pb-loaded SnS_2/DMA** and b) comparison of the N 1s spectrum of **SnS_2/DMA** and **SnS_2/acid.** S14

Table S1. Mössbauer parameters as resulting from the best fits of the corresponding spectra for the denoted samples recorded at 80 K. IS is the isomer shift relative to SnO_2 at room temperature, Γ/2 is the half linewidth, QS is the quadrupole splitting and Area is the absorption area of the components used to fit the spectra. Typical errors are ±0.02 mm/s for IS, Γ/2, and QS and ± 5% for Area. S15

Figure S17. FE-SEM images of **SnS_2/DMA PMMA@Cotton Fabric**. S15

Figure S18. EDS spectrum of **SnS_2/DMA PMMA@Cotton Fabric**. S16

Figure S19. FE-SEM images of **SnS_2/acid PMMA@Cotton Fabric**. S16

Figure S20. EDS spectrum of **SnS_2/acid PMMA@Cotton Fabric**. S17

Figure S21. Kinetics of Pb^{2+} sorption for **SnS_2/DMA** (initial Pb concentration 81.8 ppm, pH ~ 5). S17

Figure S22. Kinetics of Pb^{2+} sorption for **SnS_2/acid** (initial Pb concentration 81.8 ppm, pH ~ 5). S18

Figure S23. Kinetics of Pb^{2+} sorption for **SnS_2** (initial Pb concentration 81.8 ppm, pH ~ 5). S18

Figure S24. Isotherm Pb^{2+} sorption data for **SnS_2**. The red line represents the fitting of the data with the Langmuir model (R^2= 0.67, q_e= 250.04 ± 31.73 mg g^{-1} and b= 0.144 ± 0.177 L mg^{-1} (contact time, t= 24 h). S19

Figure S25. Percentage (%) sorption of Pb^{2+} in the pH range of 3-7 by **SnS_2/DMA** (Initial concentration of Pb^{2+}= 1 ppm, contact time = 10 min) S19

Figure S26. Percentage (%) sorption of Pb^{2+} in the pH range of 3-7 by **SnS_2/acid** (Initial concentration of Pb^{2+}= 1 ppm, contact time = 10 min) S20

Figure S27. ^{1}HNMR spectra of a) **SnS_2/DMA** digested in D_2O/HNO_3 and b) the solution after Pb^{2+} sorption ($C_{initial}$ of Pb^{2+} ~600 ppm) with **SnS_2/DMA** in D_2O S20

Figure S28. EDS spectrum of **Pb-loaded SnS_2/DMA** ($C_{initial}$ of Pb^{2+} = 10 ppm). .. S21

Figure S29. EDS spectrum of **Pb-loaded SnS_2/acid** ($C_{initial}$ of Pb^{2+} = 50 ppm). S21

Figure S30. High-resolution XPS spectra of a) S $2p_{1/2}$ and $2p_{3/2}$ (162.5 and 161.3 eV), b) Sn $3d_{3/2}$ and $3d_{5/2}$ (495.0 and 486.5 eV), c) Pb $4f_{5/2}$ and $4f_{7/2}$ (142.7 and 137.8 eV) of **Pb-loaded SnS_2/acid**, d) S $2p_{1/2}$ and $2p_{3/2}$ (162.4 and 161.2 eV), e) Sn $3d_{3/2}$ and $3d_{5/2}$ (494.7 and 486.2 eV), f) N 1s (401.5 and 399.8 eV) g) Pb $4f_{5/2}$ and $4f_{7/2}$ (142.2 and 137.5 eV) of **Pb-loaded SnS_2/DMA**. S22

Table S2. Comparison of the **Pb^{2+} batch sorption** properties of the new SnS_2-based materials with those of other sorbents S23

Table S3. Comparison of the **Pb^{2+} column sorption** properties of the new SnS_2-based materials with those of other sorbents S25

References S26

Experimental Section

Materials: Tin powder, Tin (granular), and Sulfur powder (99.9%) were purchased from Fluka AG, Sigma-Aldrich, and Merk, respectively. Dimethylamine (DMA) (40% aqueous solution) and concentrated HCl were bought from Sigma-Aldrich. Poly(methyl methacrylate) (PMMA, $(C_5H_8O_2)_n$) and sea sand were purchased from Alfa Aesar and Lachner, respectively. The solvents were used without any further treatment. Cotton fabric and bottled water were purchased from local stores.

Material Characterization

The powder X-ray diffraction patterns were recorded on a Bruker D2 Phaser X-ray diffractometer (CuKα radiation, wavelength=1.54184 Å). The variable-temperature powder X-ray diffraction patterns were recorded on a Bruker D8 Advanced X-ray diffractometer with an XRK900 in situ chamber using CuKα radiation (λ = 1.5406 Å), and a secondary monochromator operating (36 kV, 36 mA). During these experiments, inert nitrogen gas (P = 1 bar) was introduced into the chamber to monitor the sample's integrity (**SnS_2/DMA**). Before the experiment, the XRK900 reactor was purged using an Edwards oil-sealed rotary vane vacuum pump (P = 3×10^{-6} bar) to allow the circulation of N_2 gas into the chamber. Scanning Electron Microscopy (SEM) and Energy Dispersive Spectroscopy (EDS) studies were performed with a Phenom Pharos G2 Desktop FEG-SEM (Thermo Fisher Scientific) integrated with an EDS detector on Cr sputtered specimens (Q150T ES Plus automatic sputter coater, Quorum Technologies Ltd.). Before analysis, the samples were sputter-coated with a 5 nm Cr film to reduce charging. The ground samples' UV-Vis diffuse reflectance spectra were collected using a Shimadzu UV-2600i spectrophotometer equipped with an ISR-2600Plus integrating sphere in the 190-1400 cm^{-1} wavelength range. $BaSO_4$ powder was used as a reference and base material on which the powder sample was coated. The reflectance data were converted to absorption data using the Kubelka–Munk equation, and the band gap energy of the material was calculated using Tauc Plot (Equation 1) for indirect semiconductors. ATR-IR spectra were recorded in the 4000-400 cm^{-1} range using an Agilent Cary 630 FTIR photometer using diamond ATR. Zeta potential measurements were performed with a Malvern Zetasizer Nano ZS (Malvern Panalytical, Worcestershire, UK) in a two-electrode capillary cell. Thermal analysis was conducted from 20 to 580 °C under N_2, in a STA 449C JURITER NETZSCH, with a heating rate of 10 °C min^{-1}. The residual concentration of Pb^{2+} was determined by flame (acetylene) and graphite furnace atomic absorption spectrometry (ETAAS) with a hollow cathode lamp operating at 12 mA (Shimadzu AA-6800 atomic absorption spectrophotometer, Shimadzu Corp., Kyoto, Japan). X-ray photoelectron Spectroscopy (XPS) measurements were performed using a surface analysis ultrahigh vacuum system (SPECS GmbH) equipped with a twin Al-Mg anode X-ray source and a multichannel

hemispherical sector electron analyzer (HAS-Phoibos 100). The base pressure during the analysis was maintained at 2-5 x 10^{-9} mbar. A monochromatized Mg Kα radiation source with an energy of 1253.6 eV and a pass energy of 20 eV for the analyzer was utilized for all XPS measurements. Binding energies were determined by referencing the C 1s peak of carbon at 284.5 eV. XRF measurements were performed using an energy-dispersive micro-XRF spectrometer (Bruker M1-Mistral) equipped with a high-energy-resolution silicon drift detector (SDD) and a micro-focus X-ray W tube, operating at 50 kV voltage and 800 μA current. ^{119}Sn Mossbauer spectra of the powder samples were collected at 80 K in transmission geometry, using a constant-acceleration Mössbauer spectrometer equipped with a $Ca^{119m}SnO_3$ source kept at RT and a variable-temperature (Thor Cryogenics) liquid nitrogen bath cryostat. The spectrometer was calibrated with metallic iron at 80 K and the analyses of the spectra were performed by the IMSG code using Lorentzian-type lines.[1] ^{1}H NMR spectra were measured with a Bruker 250 MHz spectrometer.

Methods

Calculation of the band gap energy

The Tauc plot method[2] assumes that the absorption coefficient (α) can be expressed by the following equation:

$$(a * hv)^{1/\gamma} = B(hv - E_g) \quad (1)$$

where h is the Plank constant, v is the photon's frequency, E_g is the band gap energy, and B is a constant. The γ factor depends on the nature of the electron transition and is equal to $1/2$ or 2, for direct or indirect transition band gaps, respectively.

Isolation of the Pb-loaded materials

The **Pb-loaded SnS_2/DMA** and **SnS_2/acid** were isolated as follows: 10 mg of the MS were added as a solid to a solution of Pb^{2+} (10 and 50 ppm, respectively) in deionized water (10 mL, pH =5.5). The mixture was kept under vigorous magnetic stirring for approximately 30 min. The metal sulfide material was isolated by centrifugation, washed with water and acetone a few times, and dried at 80 ℃ overnight.

Batch sorption studies

Kinetic studies

Pb^{2+} sorption experiments of various reaction times (1 min - 24 h) were conducted for the sorption kinetics study. For each experiment, 10 mg of the sorbent were added to a 10 mL solution of Pb^{2+} with the desired concentration, and the mixtures were kept under magnetic stirring for the designated reaction times. The suspensions from the various reaction times were filtrated, and the resulting solutions were analyzed for their Pb^{2+} content using atomic absorption spectroscopy using a graphite chamber or flame (acetylene). Lagergren's First-order equation[3] was used to fit the kinetics data (Equation 2). The expression of the equation is the following:

Lagergren's First-order equation: $q_t = q_e[1 - \exp(-K_L t)]$
(2)

where q_e = the amount (mg g^{-1}) of ion sorbed in equilibrium, K_L = the Lagergren's or first-order rate constant.

Isotherm study

The Pb^{2+} sorption from solutions of various concentrations was studied by the batch method at V:m ~ 1000 mL/g, room temperature, for 10 min and 24 h contact. These data were used to determine Pb^{2+} isotherms. The solutions were analyzed by flame with atomic absorption spectroscopy. The Langmuir[4] and Langmuir-Freundlich[5] isotherm models[6] (Equations 3 and 4) were applied to simulate isotherm sorption data. The expressions of the models are the following:

Langmuir: $$q = q_m \frac{bC_e}{1+b\,C_e} \quad (3)$$

Langmuir-Freundlich: $$q = q_m \frac{(bC_e)^n}{1+(bC_e)^n} \quad (4)$$

where q (mg g^{-1}) is the amount of the anion sorbed at the equilibrium concentration C_e (ppm), q_m is the maximum sorption capacity of the sorbent, b (L mg^{-1}) is the Langmuir constant related to the free energy of the sorption and n is the Freundlich constant.

Competitive sorption and pH studies

Lastly, the competitive, bottled water and variable pH Pb^{2+} sorption experiments were carried out with the batch method at V: m ratio (1000) mL/g, room temperature, and 10 min contact.

Sorption under continuous flow conditions

Preparation of the column: Before the sorption studies, the MS-PMMA@Cotton Fabrics were immersed and left to soak in aqueous HCl (1M) overnight. This pretreatment aimed at activating the sorbents via acid-induced Sn leaching that can increase the Sn deficiency and the surface negative charge of the materials. The next day, the MS-PMMA@Cotton Fabrics (x6) were washed with deionized water and packed inside a glass column (1.5 cm x 30 cm) divided by sea sand (Figure 7). The column was washed with 150 mL of deionized H_2O to remove impurities from the sand.

Column studies: **SnS_2/DMA@PMMA Cotton Fabric** and **SnS_2/acid@PMMA Cotton Fabric**, used in the column sorption studies, contained approximately 16.6 and 17.1 mg of the sorbent, respectively. The Pb^{2+} contaminated bottled water solution was pumped through the column in down-flow mode (flow rate of 0.8 mL/min). A total number of 14 bed volumes were collected for every run, and the concentration of the residual Pb^{2+} was analyzed with atomic absorption spectroscopy. The column containing sea sand and PMMA@Cotton fabrics (without MS) was also tested for Pb^{2+} sorption under continuous flow conditions, showing no Pb^{2+} sorption capacity.

The **breakthrough capacity**[7] of the column can be determined by the equation:

$$Q_b = C_0 V_b \tag{5}$$

where C_0 is the initial Pb^{2+} concentration (mg L^{-1}) and V_b is the volume (L) passed until the breakpoint concentration.

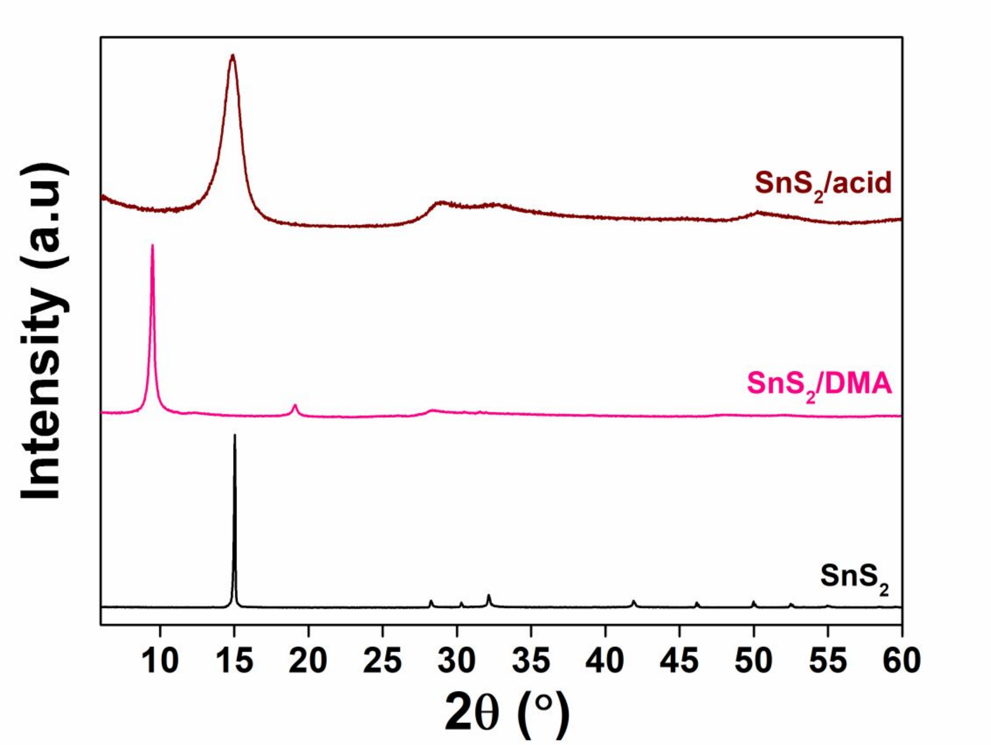

Figure S1. PXRD patterns of **SnS_2** (black), **SnS_2/DMA** (pink) and **SnS_2/acid** (wine).

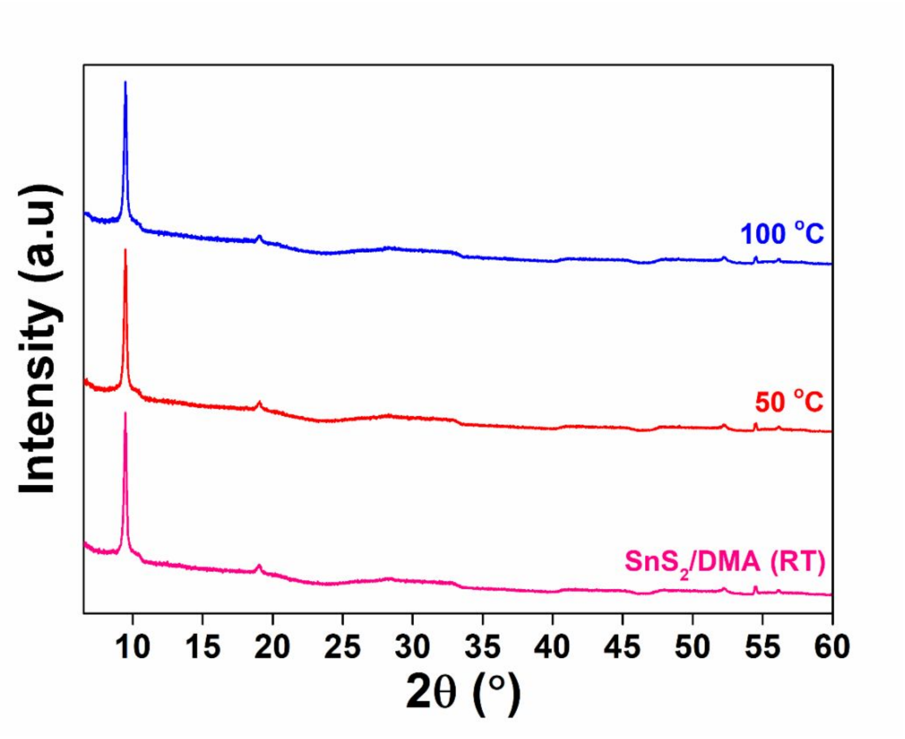

Figure S2. VT-PXRD patterns of **SnS_2/DMA** at room temperature (RT), 50 and 100℃.

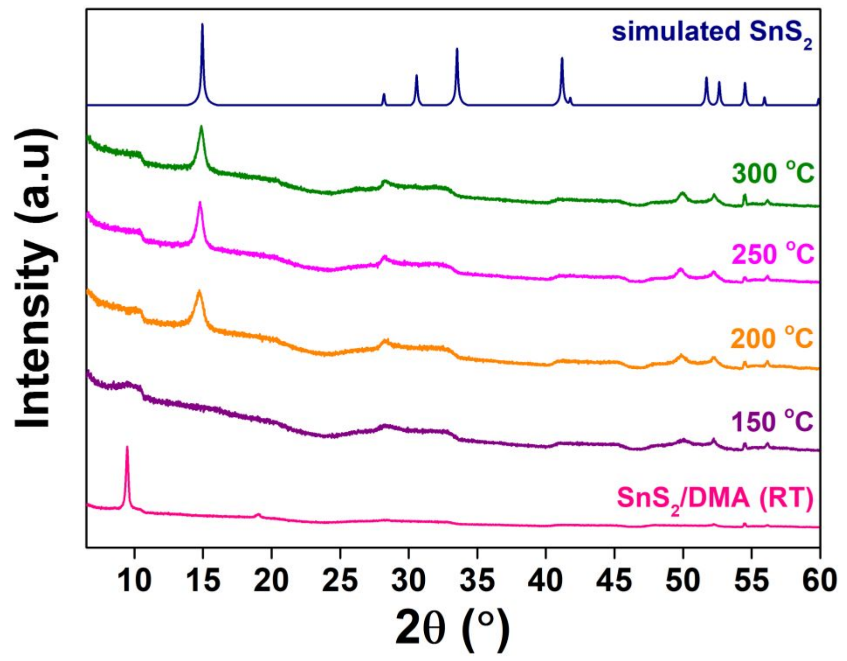

Figure S3. VT-PXRD patterns of **SnS_2/DMA** at room temperature (RT), 150-300 ℃ and the simulated pattern of SnS_2.

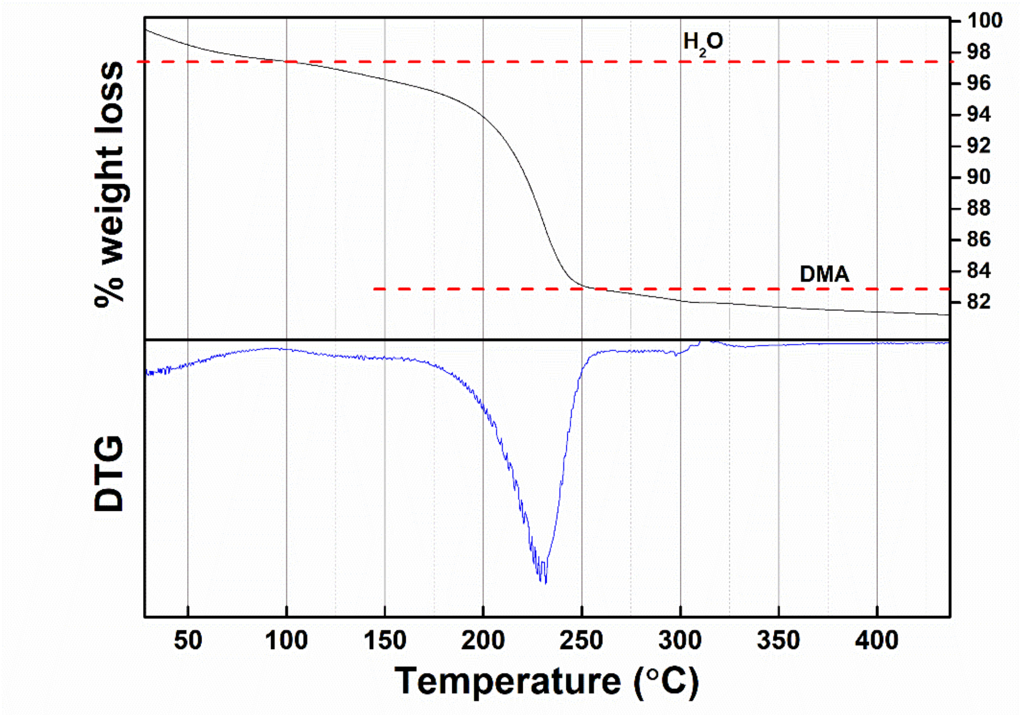

Figure S4. TGA data of **SnS_2/DMA** measured in N_2 atmosphere.

The **SnS_2/DMA** composition can be written as $SnS_2(DIMA)_x(H_2O)_y$. The TGA data (Figure S4) reveal a weight loss of 2.6 % from 35 to 102 ℃, assigned to removing H_2O, and a second weight loss of 14.6 % from 102 to 250 ℃, attributed to the release of dimethylamine. From these results, the water and dimethylamine content are calculated as follows:

A. 100 g of **SnS_2/DMA** release 2.6 and 14.6 g of H_2O and DMA, respectively
B. (182.81 + 45.08*x + 18.015*y) contain (45.08*x) g of DMA and (18.015*y) g of H_2O

The molecular weight of $SnS_2(DIMA)_x(H_2O)_y$ can be calculated as (182.81 + 45.08*x + 18.015*y).

From the above, x was found to be ~ 0.71, which responds to 0.71 mol of DMA/mol of SnS_2/DMA, and y was found to be ~0.32, which responds to 0.32 mol of H_2O/mol of **SnS_2/DMA**.

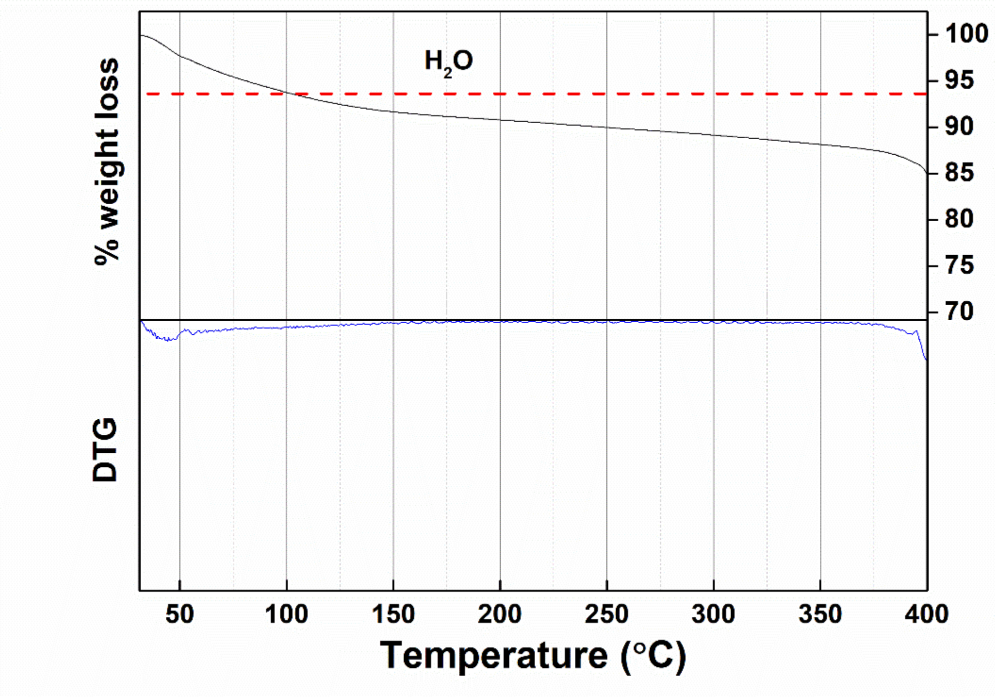

Figure S5. TGA data of **SnS_2/acid** measured in N_2 atmosphere.

The TGA data for **SnS_2/acid** (Figure S5) reveal a weight loss of 6.3 % from 35 to 106 ℃, assigned to removing H_2O. No other weight loss is observed at higher temperatures, meaning dimethylamine is absent from **SnS_2/acid**. The **SnS_2/acid** composition can be written as $Sn_{1-x}S_2{\cdot}yH_2O$, x≤0.1, since the Sn deficiency is too small to be determined considering EDS and XRF measurements' accuracy (5-10%). Thus, the composition can be simplified as $SnS_2{\cdot}yH_2O$. From these results, the water content is calculated as follows:

A. 100 g of **SnS_2/acid** release 6.3 g of H_2O

B. (182.81 + 18.015*y) contain (18.015*y) g of H_2O

The molecular weight of $SnS_2{\cdot}yH_2O$ can be calculated as (182.81 + 18.015*y).

From the above, y was found to be 0.68, which responds to 0.68 mol of H_2O/mol of **SnS_2/acid**.

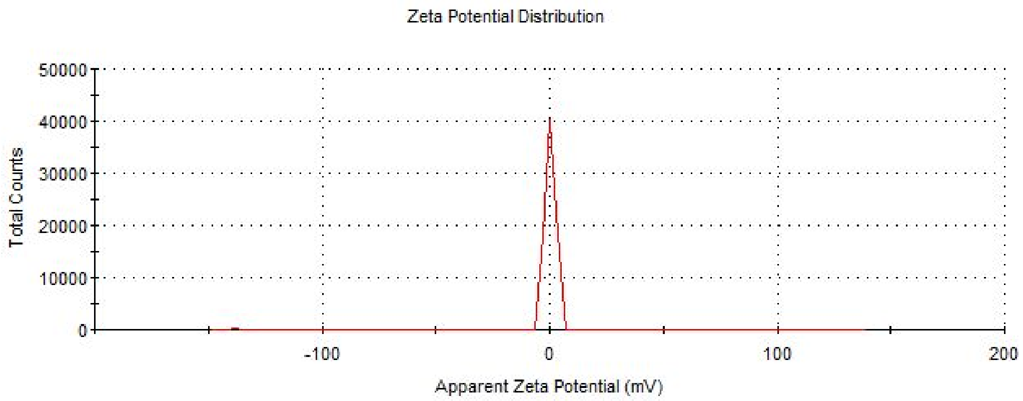

Figure S6. Zeta-potential diagram of **SnS_2/DMA**.

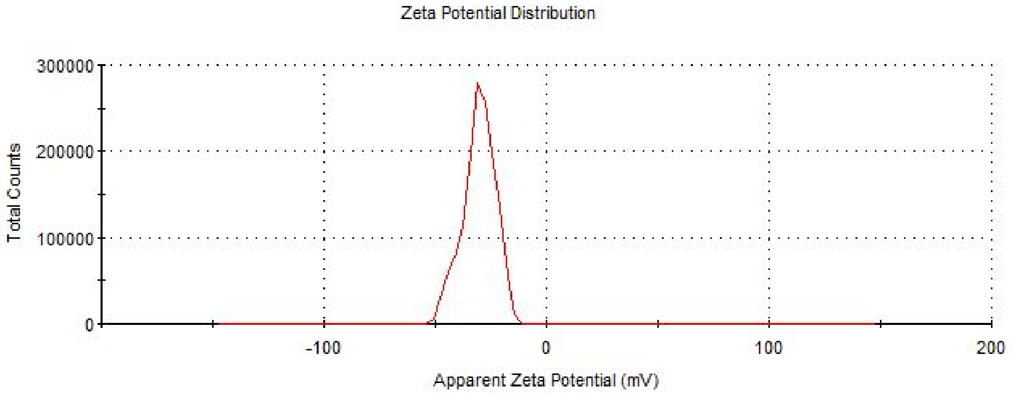

Figure S7. Zeta-potential diagram of **SnS_2/acid**.

	Element	AN	Net sum	norm. C. [wt.%]	Atom C. [at.%]
	Tin	50	679735	66.01	34.41
	Argon	18	25890	0.00	0.00
	Rhodium	45	328047	0.00	0.00
	Sulfur	16	642792	33.99	65.59
			Total	100.00	100.00

Figure S8. XRF measurements of **SnS_2/acid**.

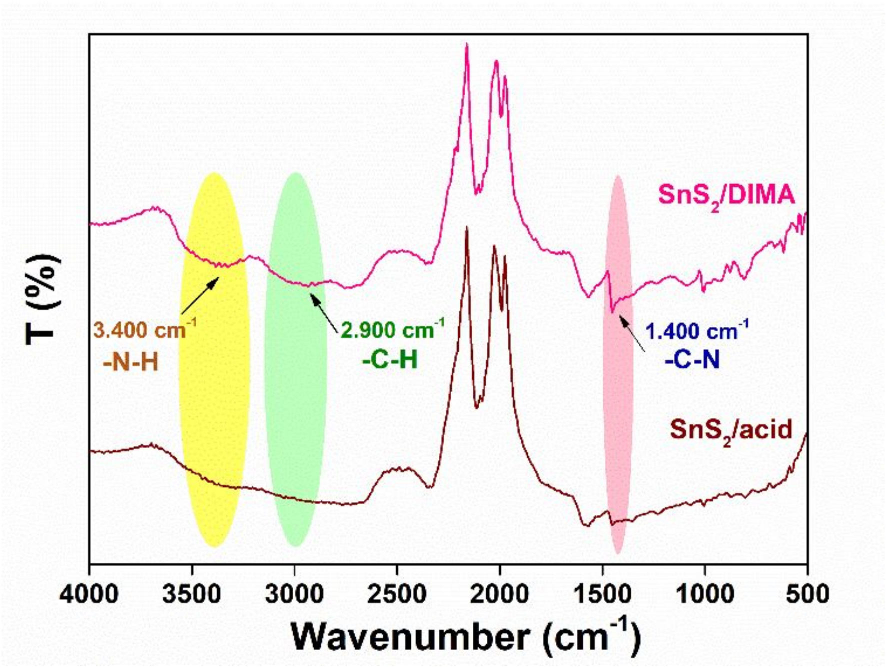

Figure S9. IR-spectra of **SnS_2/DMA** (pink) and **SnS_2/acid** (wine). The peaks around 2000 cm^{-1} are attributed to the diamond ATR.[8]

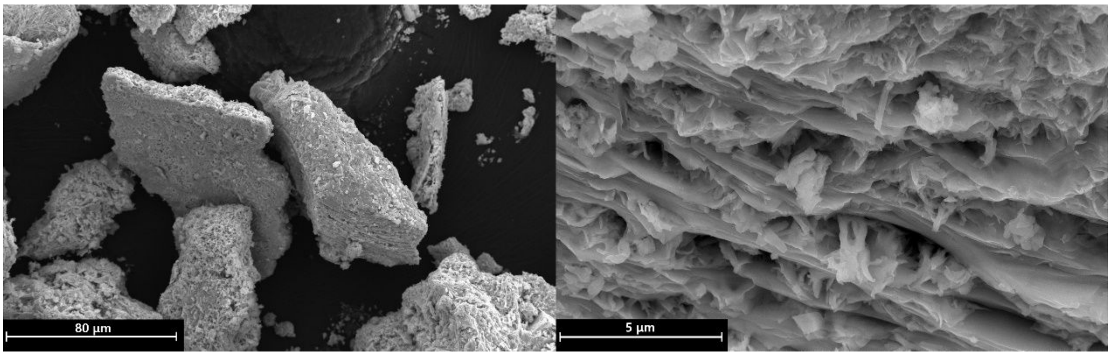

Figure S10. FE-SEM images of **SnS_2/DMA**.

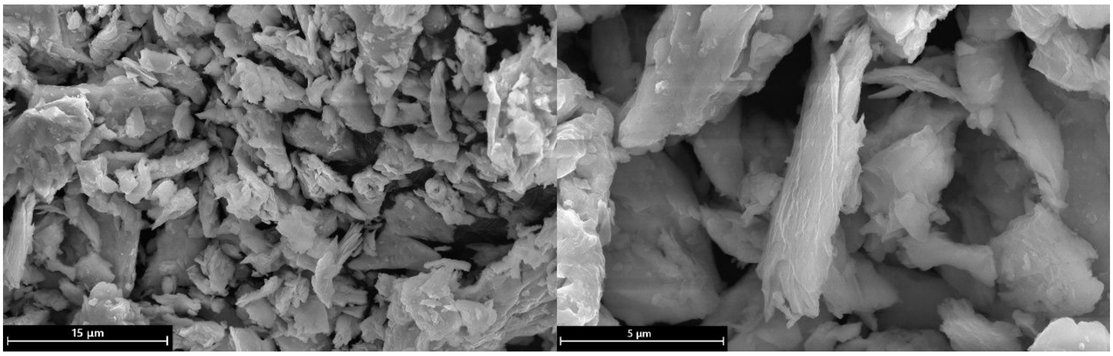

Figure S11. FE-SEM images of **SnS_2/acid**.

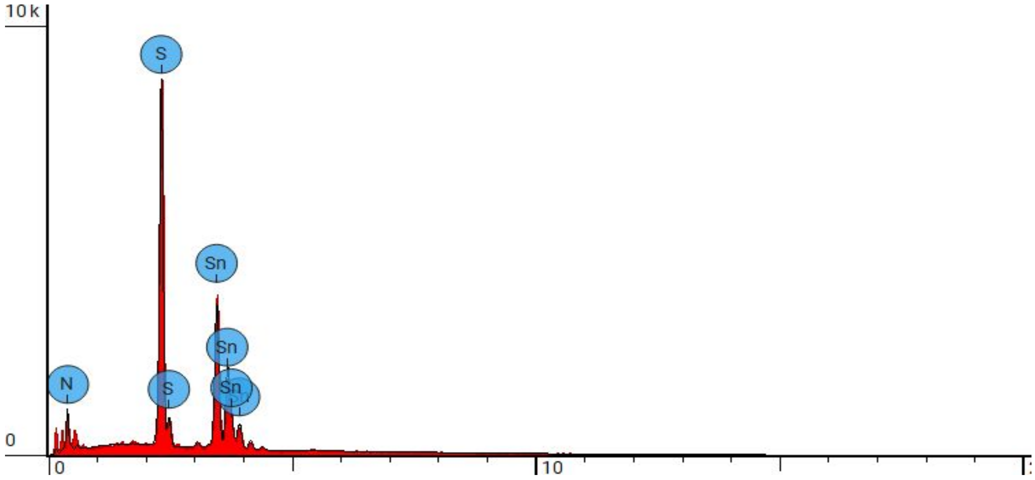

Figure S12. EDS spectrum of $\mathbf{SnS_2}$**/DMA**.

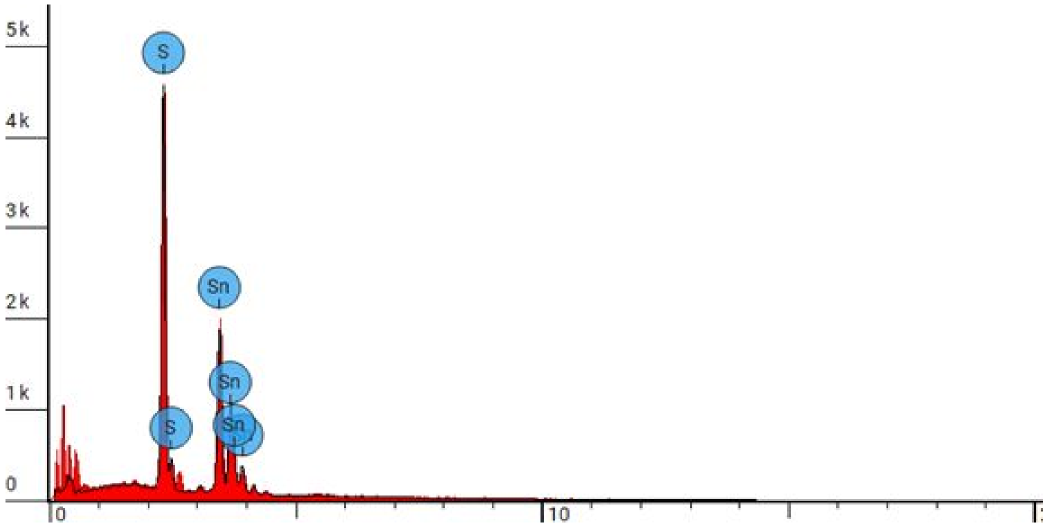

Figure S13. EDS spectrum of $\mathbf{SnS_2}$**/acid**.

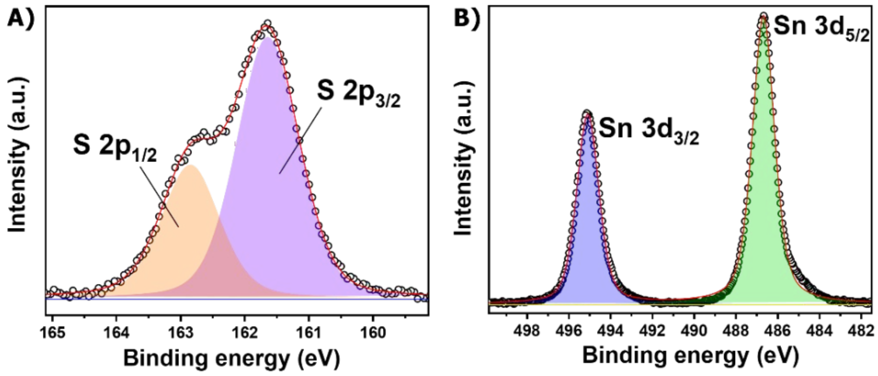

Figure S14. High-resolution XPS spectra of a) S $2p_{1/2}$ and $2p_{3/2}$ and b) Sn $3d_{3/2}$ and $3d_{5/2}$ of pristine $\mathbf{SnS_2}$.

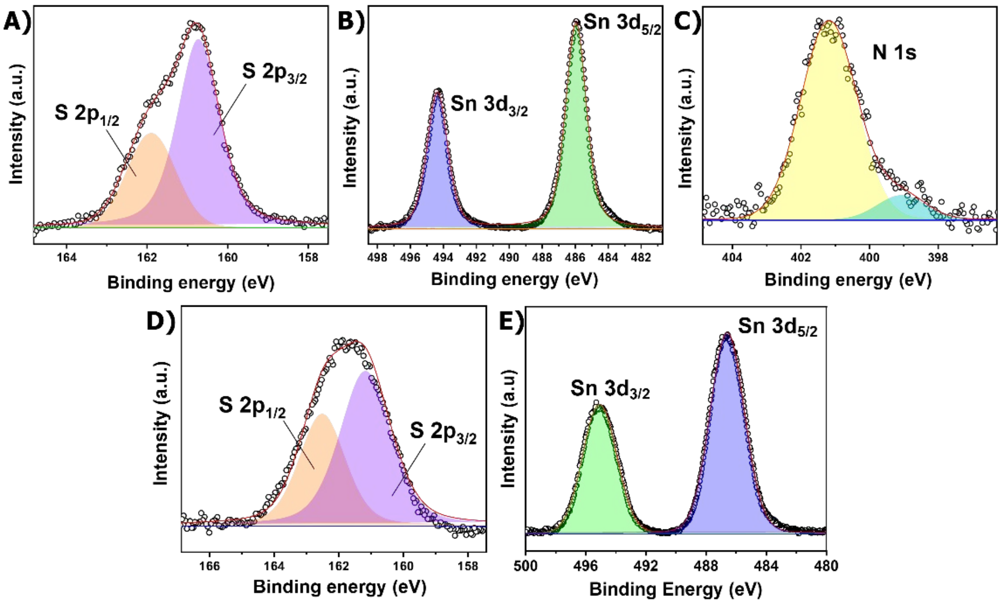

Figure S15. High resolution XPS spectra of a) S $2p_{1/2}$ and $2p_{3/2}$ b) Sn $3d_{3/2}$ and $3d_{5/2}$ and c) N 1s of **SnS_2/DMA**, d) S $2p_{1/2}$ and $2p_{3/2}$ and e) Sn $3d_{3/2}$ and $3d_{5/2}$ of **SnS_2/acid**.

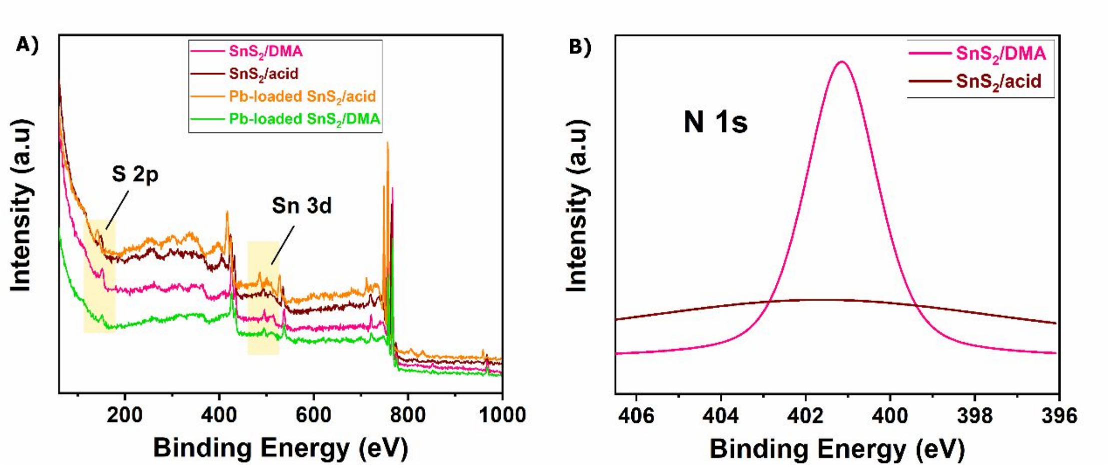

Figure S16. a) XPS survey of **SnS_2/DMA**, **SnS_2/acid**, **Pb-loaded SnS_2/acid** and **Pb-loaded SnS_2/DMA** and b) comparison of the N 1s spectrum of **SnS_2/DMA** and **SnS_2/acid.**

Table S1. Mössbauer parameters as resulting from the best fits of the corresponding spectra for the denoted samples recorded at 80 K. IS is the isomer shift relative to SnO_2 at room temperature, Γ/2 is the half linewidth, QS is the quadrupole splitting and Area is the absorption area of the components used to fit the spectra. Typical errors are ±0.02 mm/s for IS, Γ/2, and QS and ± 5% for Area.

Sample	IS	Γ/2	QS	Area	Assignement
	mm/s	mm/s	mm/s	%	
**pristine SnS_2**	1.06	0.58	0.00	100	Sn^{4+} at regular octahedra
**SnS_2/DMA**	1.04	0.48	0.00	54	Sn^{4+} at regular octahedra
	1.11	0.48	0.94	46	Sn^{4+} at distorted octahedra
**SnS_2/acid**	1.03	0.52	0.00	50	Sn^{4+} at regular octahedra
	1.01	0.52	1.04	50	Sn^{4+} at distorted octahedra
**Pb-loaded SnS_2/acid**	0.97	0.60	0.00	53	Sn^{4+} at regular octahedra
	0.77	0.60	1.27	47	Sn^{4+} at distorted octahedra

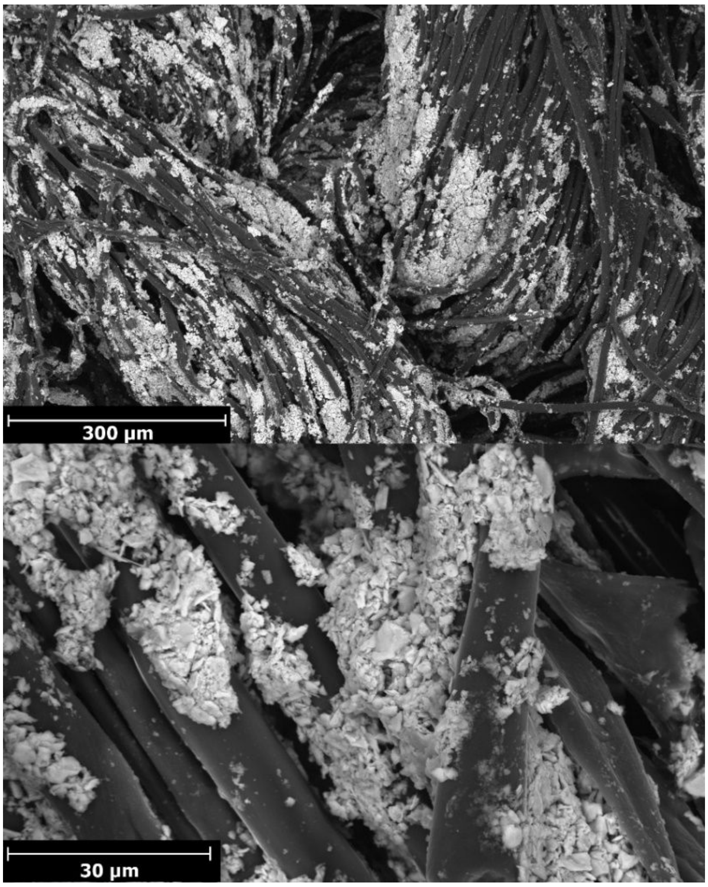

Figure S17. FE-SEM images of **SnS_2/DMA PMMA@Cotton Fabric**.

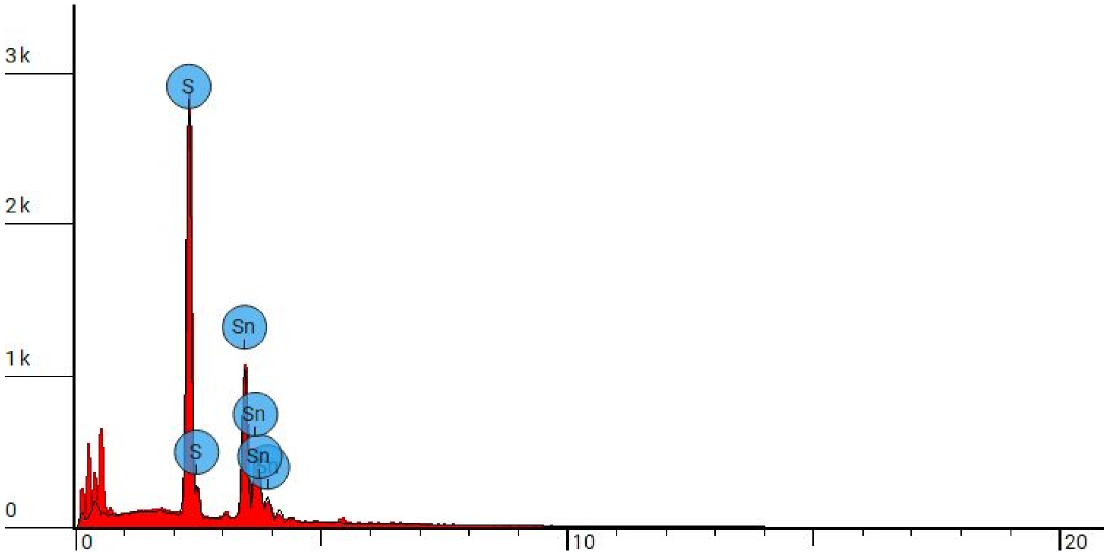

Figure S18. EDS spectrum of **SnS_2/DMA PMMA@Cotton Fabric**.

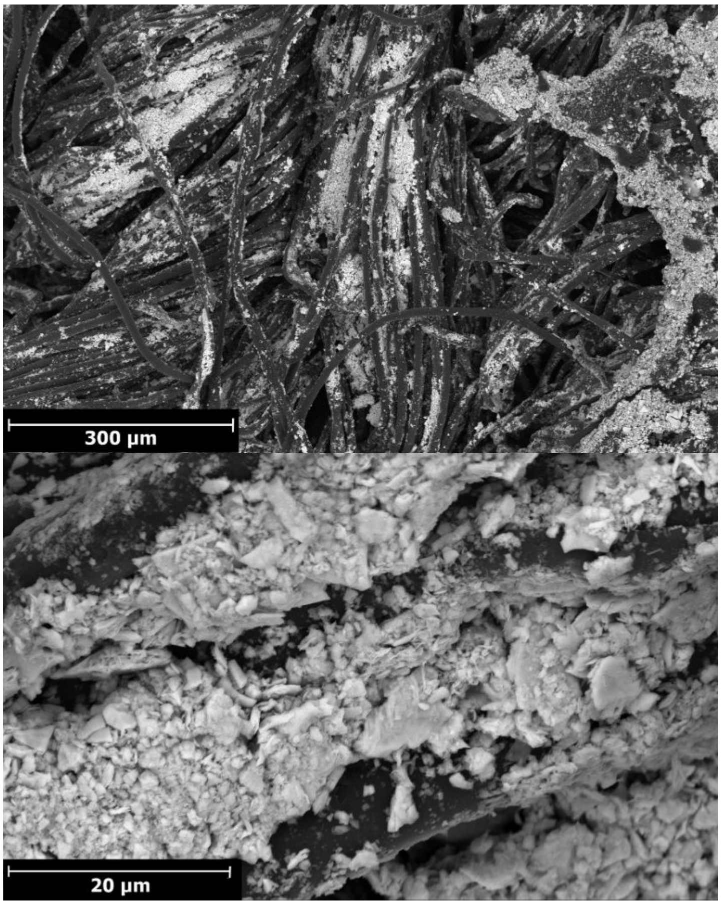

Figure S19. FE-SEM images of **SnS_2/acid PMMA@Cotton Fabric**.

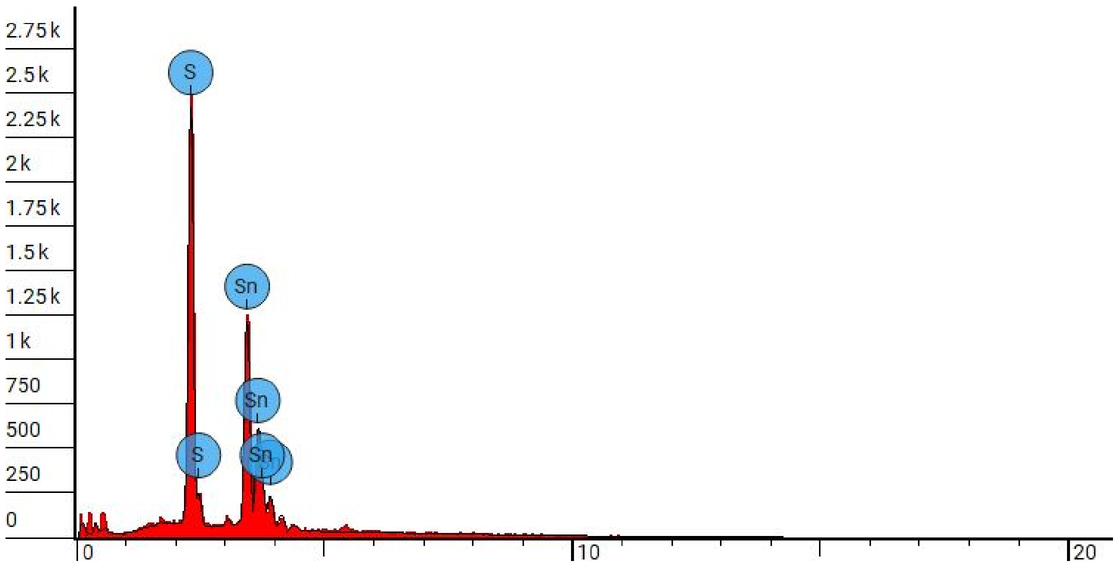

Figure S20. EDS spectrum of **SnS_2/acid PMMA@Cotton Fabric**.

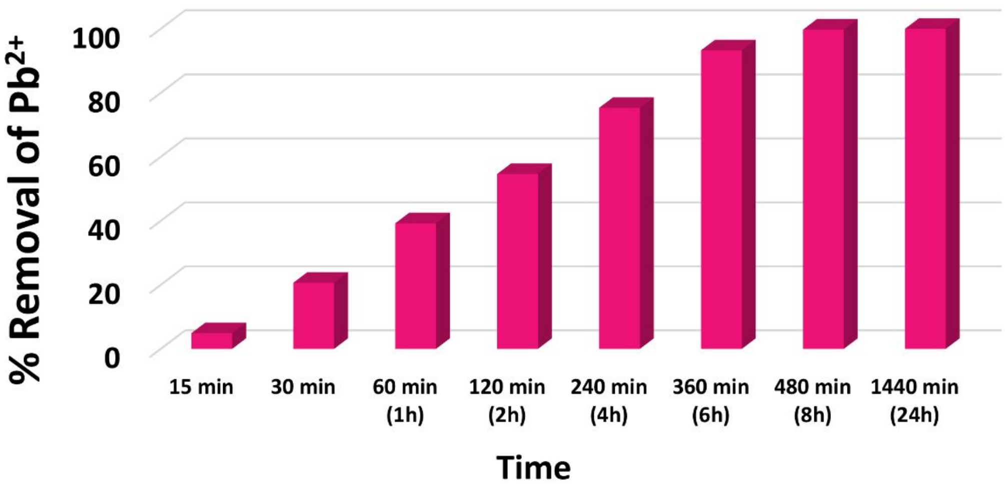

Figure S21. Kinetics of Pb^{2+} sorption for **SnS_2/DMA** (initial Pb concentration 81.8 ppm, pH ~ 5).

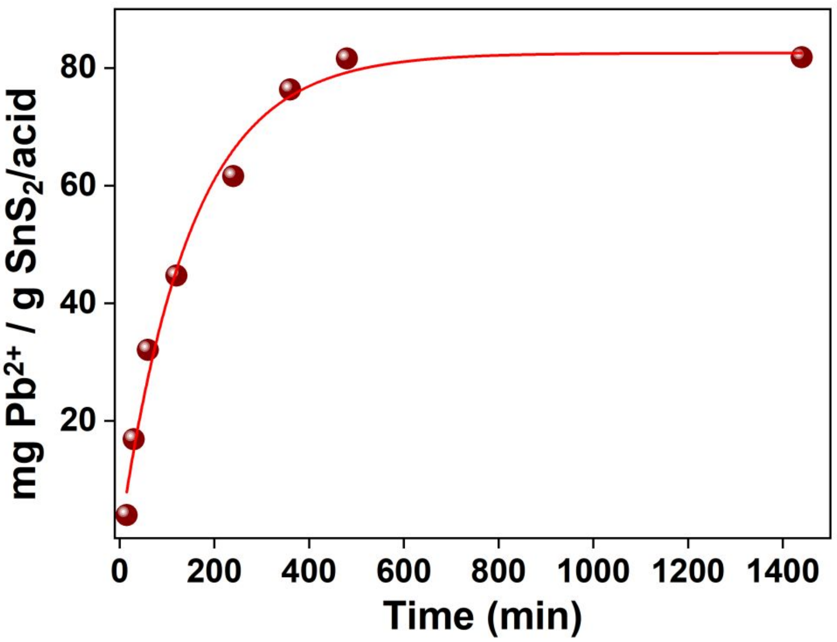

Figure S22. Kinetics of Pb^{2+} sorption for **SnS_2/acid** (initial Pb concentration 81.8 ppm, pH ~ 5).

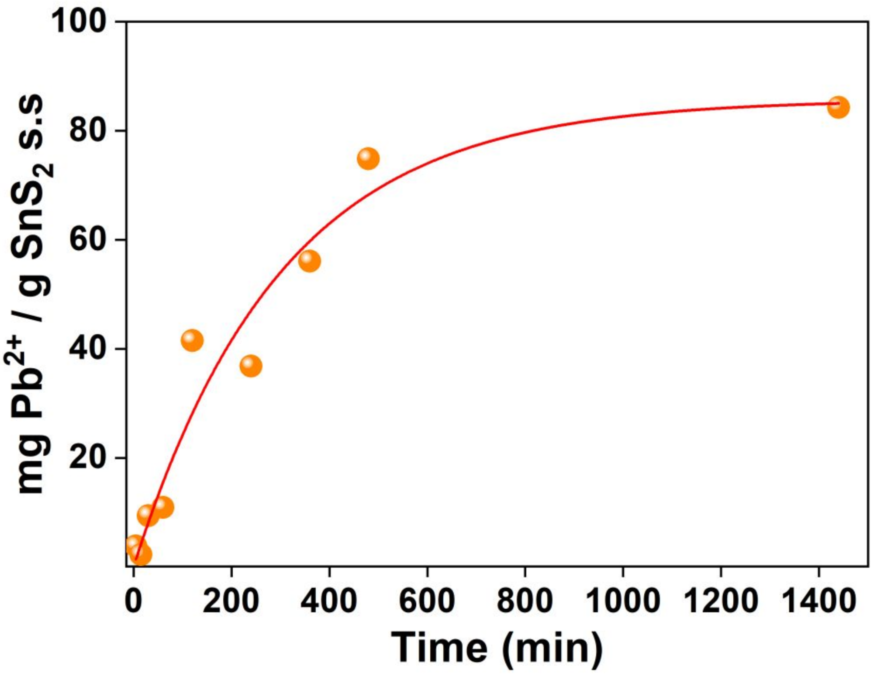

Figure S23. Kinetics of Pb^{2+} sorption for **SnS_2** (initial Pb concentration 81.8 ppm, pH ~ 5).

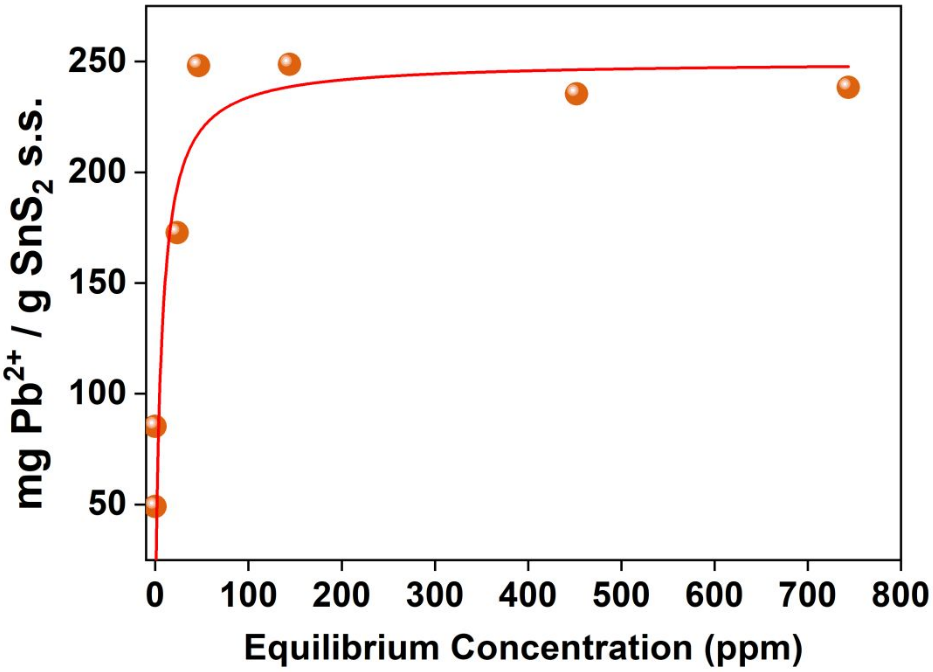

Figure S24. Isotherm Pb^{2+} sorption data for **SnS_2**. The red line represents the fitting of the data with the Langmuir model (R^2= 0.67, q_e= 250.04 ± 31.73 mg g^{-1} and b= 0.144 ± 0.177 L mg^{-1} (contact time, t= 24 h).

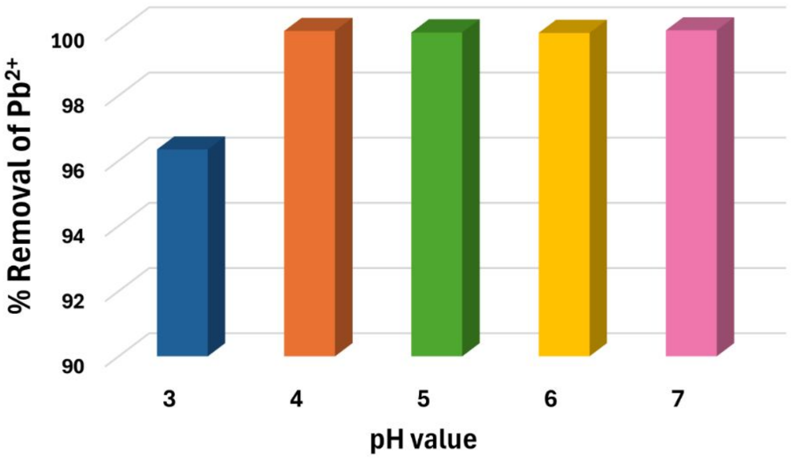

Figure S25. Percentage (%) sorption of Pb^{2+} in the pH range of 3-7 by **SnS_2/DMA** (Initial concentration of Pb^{2+}= 1 ppm, contact time = 10 min).

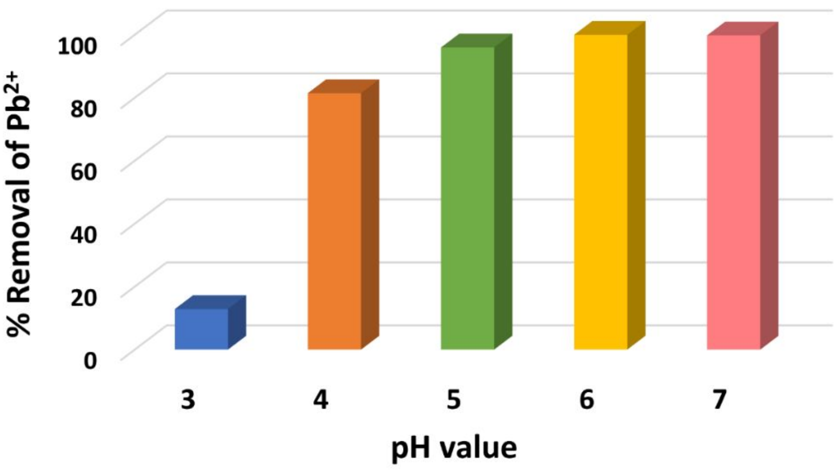

Figure S26. Percentage (%) sorption of Pb^{2+} in the pH range of 3-7 by **SnS_2/acid** (Initial concentration of Pb^{2+}= 1 ppm, contact time = 10 min).

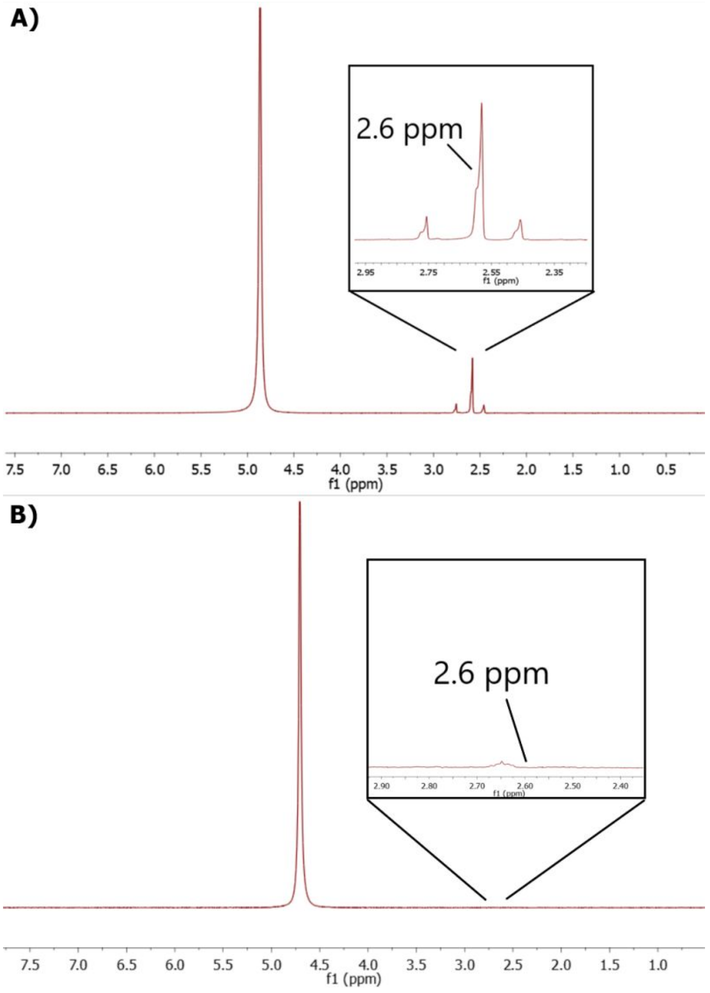

Figure S27. ^{1}HNMR spectra of a) **SnS_2/DMA** digested in D_2O/HNO_3 and b) the solution after Pb^{2+} sorption ($C_{initial}$ of Pb^{2+} ~600 ppm) with **SnS_2/DMA** in D_2O.

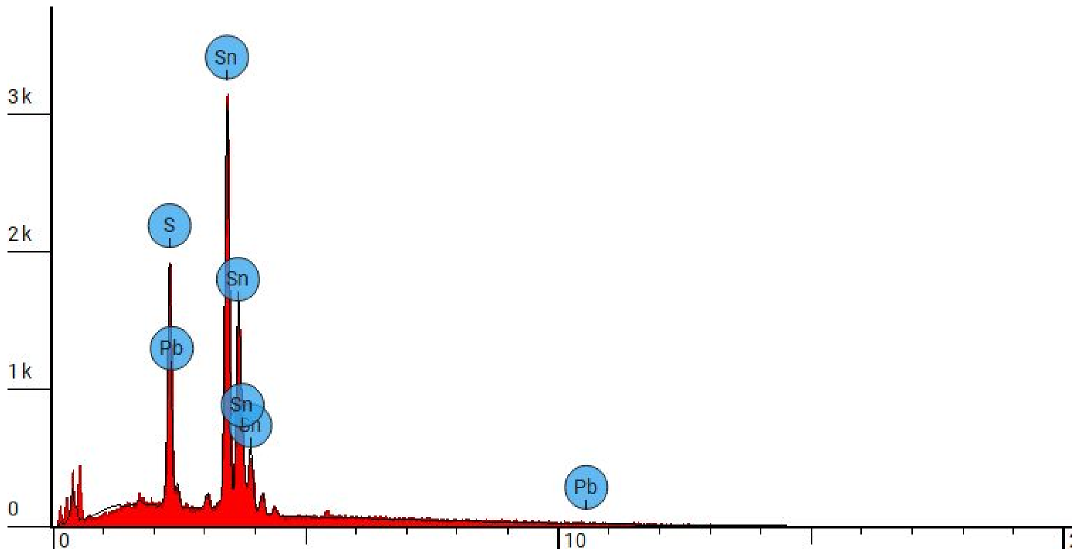

Figure S28. EDS spectrum of **Pb-loaded SnS_2/DMA** ($C_{initial}$ of Pb^{2+} = 10 ppm).

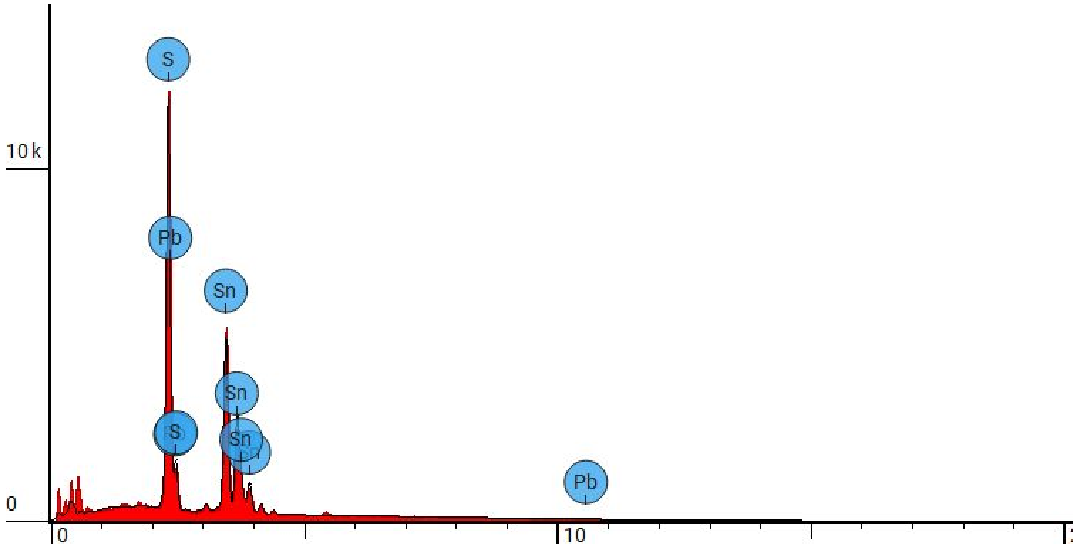

Figure S29. EDS spectrum of **Pb-loaded SnS_2/acid** ($C_{initial}$ of Pb^{2+} = 50 ppm).

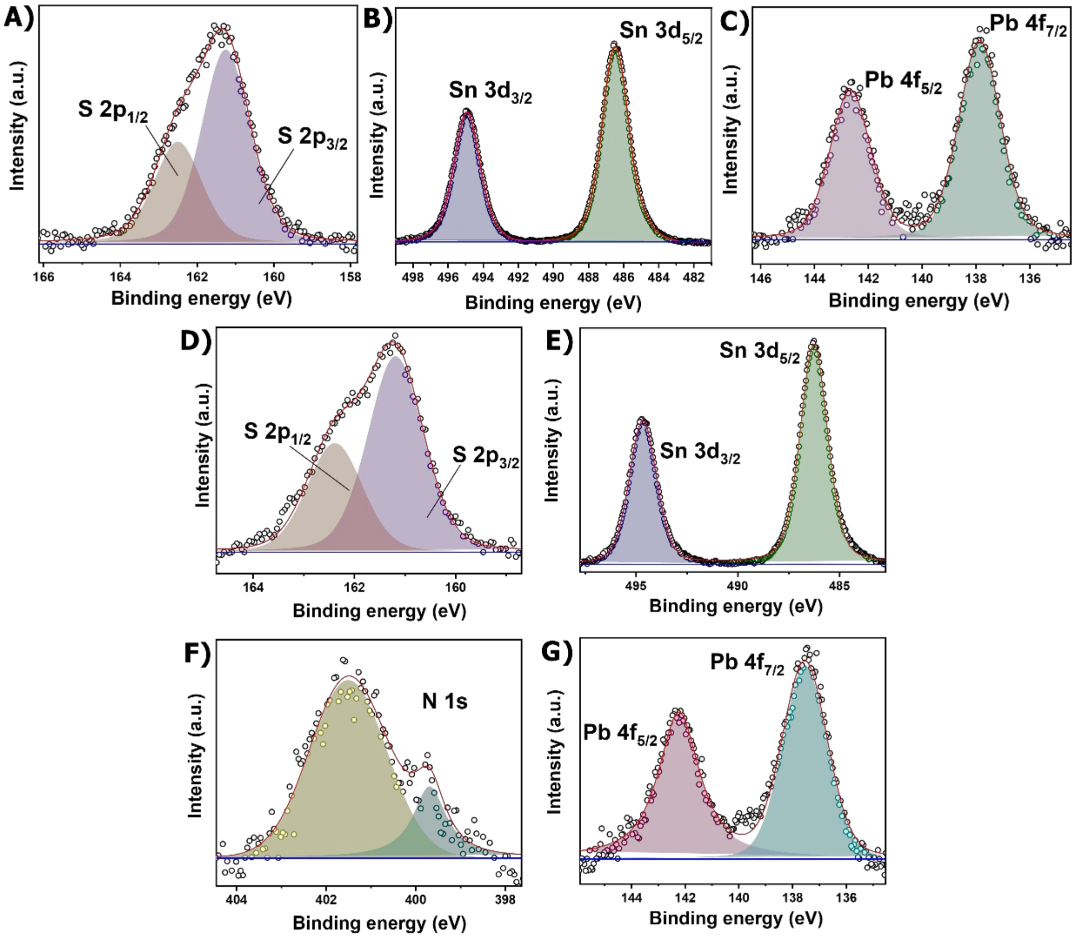

Figure S30. High-resolution XPS spectra of a) S $2p_{1/2}$ and $2p_{3/2}$ (162.5 and 161.3 eV), b) Sn $3d_{3/2}$ and $3d_{5/2}$ (495.0 and 486.5 eV), c) Pb $4f_{5/2}$ and $4f_{7/2}$ (142.7 and 137.8 eV) of **Pb-loaded SnS_2/acid**, d) S $2p_{1/2}$ and $2p_{3/2}$ (162.4 and 161.2 eV), e) Sn $3d_{3/2}$ and $3d_{5/2}$ (494.7 and 486.2 eV), f) N 1s (401.5 and 399.8 eV) g) Pb $4f_{5/2}$ and $4f_{7/2}$ (142.2 and 137.5 eV) of **Pb-loaded SnS_2/DMA**.

Table S2. Comparison of the $\mathbf{Pb^{2+}}$ **batch sorption** properties of the new SnS_2-based materials with those of other sorbents.

Sorbent	Capacity mg/g	Equilibrium time	Selectivity	Reusability	Reference
KMS-1	377	5 min	vs. alkali and alkaline earth cations	Not reusable	5
MoS_4-LDH	290	30 min	vs. Co^{2+}, Ni^{2+}, Zn^{2+}, Cd^{2+}	Not reusable	9
S_x -LDH	483	3 days	vs. Co^{2+}, Ni^{2+}, Zn^{2+}	Not reusable	10
CMS	1053	120 min	vs. Na^{+}, Mg^{2+}, Ca^{2+}	Not reusable	11
p-UCR-20	527	<1 min	vs. Na^{+}, Mg^{2+}, Ca^{2+}, K^{+}	Not reusable	12
A500-MoS_4 resin	495	100 min	vs. Cu^{2+}, Cd^{2+}, Hg^{2+}	Reusable	13
Ni-MOF-74	370	120 min	vs. Na^{+}	Reusable	14
Ni/Fe/Ti–MoS_4-LDH	653	80 min	vs. Ni^{2+}, Cu^{2+}, Zn^{2+}, Fe^{3+}, Pb^{2+}, Ag^{+}	Not reusable	15
LS-GH	1210	12 h	-	Reusable	16
C@MoS_2/MMT	187	480 min	vs. Cu^{2+}, Zn^{2+}, Cr^{6+}, Cd^{2+}	Not reusable	17
MoS_2/Fe_3O_4	264	180 min	vs. Cu^{2+}, Zn^{2+}, Cd^{2+}, Mg^{2+}	Not reusable	18
Na^{+}-MOR-1-$NHCS_2$	334	<1 min	vs. Na^{+}, Mg^{2+}, Ca^{2+}, K^{+}	Reusable	19

K–Co–Mo–S_x chalcogel	1146	60 min	vs. Ag^{+}, Hg^{2+}, Cu^{2+}, Ni^{2+}, Cd^{2+}	Not reusable	20
K-MPS-1	394	8 h	vs. Na^{+}, Mg^{2+}, Ca^{2+}	Not reusable	21
Ca-MOF	522	5 min	vs. Na^{+}, Mg^{2+}, Ca^{2+}, Ni^{2+}, Zn^{2+}, Cu^{2+}	Not reusable	22
**pristine SnS_2**	**250**	**24 h**	**-**	**-**	**This work**
**SnS_2/DMA**	**838**	**24 h**	**vs. Mg^{2+}, Ca^{2+}, Na^{+}, K^{+}, NH_4^{+}**	**Reusable**	**This work**
**SnS_2/acid**	**190**	**24 h**	**vs. Mg^{2+}, Ca^{2+}, Na^{+}, K^{+}, NH_4^{+}**	**Reusable**	**This work**

Table S3. Comparison of the **Pb^{2+} column sorption** properties of the new SnS_2-based materials with those of other sorbents.

Sorbent	Sorbent mass	Capacity (mg/g)	Initial Concentration	Flow rate (mL/min)	Solution	Reusability	Reference
p-UCR-20-CA	50 mg	1.9	100 ppb	1.0	wastewater simulant	Not reusable	12
Ca-MOF	50 mg	2.4	100 ppb	1.0	wastewater simulant	Not reusable	22
Ni/Mo-LDH	-	165.65	50 ppm	10.0	industrial wastewater	Reusable	23
ZSM-5/AC	270 mg	213.3	250 ppm	2.0	aqueous solution	Reusable	24
Titanosilicate ETS-10	3.35 g	348.1	1058 ppm	2.0	aqueous solution	Reusable	25
**SnS_2/DMA-PMMA@Cotton Fabric**	**16.6 mg**	**49.4 – 72.3**	**10 ppm**	**0.8**	**bottled water**	**Reusable**	**This work**
**SnS_2/acid-PMMA@Cotton Fabric**	**17.1 mg**	**29.8 – 49.7**	**10 ppm**	**0.8**	**bottled water**	**Reusable**	**This work**

References

(1) Douvalis, P.; Polymeros, A.; Bakas, T. A ^{57}Fe-^{119}Sn Mössbauer spectra computer fitting program with novel interactive user interface. *J. Phys. Conf. Ser.* **2010**, *217*, 012014.

(2) Makuła, P.; Pacia, M.; Macyk, W. How To Correctly Determine the Band Gap Energy of Modified Semiconductor Photocatalysts Based on UV-Vis Spectra. *J. Phys. Chem. Lett.* **2018**, *9*, 6814–6817.

(3) Benhammou, A.; Yaacoubi, A.; Nibou, L.; Tanouti, B. Adsorption of Metal Ions onto Moroccan Stevensite: Kinetic and Isotherm Studies. *J. Colloid Interface Sci.* **2005**, *282* (2), 320–326.

(4) Manos, M. J.; Ding, N.; Kanatzidis, M. G. Layered Metal Sulfides: Exceptionally Selective Agents for Radioactive Strontium Removal. *Proc. Natl. Acad. Sci. U. S. A.* **2008**, *105* (10), 3696–3699.

(5) Manos, M. J.; Kanatzidis, M. G. Sequestration of Heavy Metals from Water with Layered Metal Sulfides. *Chem. Eur. J.* **2009**, *15* (19), 4779–4784.

(6) Babatunde, K. A.; Negash, B. M.; Jufar, S. R.; Ahmed, T. Y.; Mojid, M. R. Adsorption of Gases on Heterogeneous Shale Surfaces: A Review. *J. Pet. Sci. Eng.* **2022**, *208*, 109466.

(7) Rapti, S.; Pournara, A.; Sarma, D.; Papadas, I. T.; Armatas, G. S.; Hassan, Y. S.; Alkordi, M. H.; Kanatzidis, M. G.; Manos, M. J. Rapid, Green and Inexpensive Synthesis of High Quality UiO-66 Amino-Functionalized Materials with Exceptional Capability for Removal of Hexavalent Chromium from Industrial Waste. *Inorg. Chem. Front.* **2016**, *3*, 697-707.

(8) Day, M. C.; Pamato, M. G.; Novella, D.; Nestola, F. Imperfections in Natural Diamond: The Key to Understanding Diamond Genesis and the Mantle. *Riv. Nuovo Cim*. **2023**, *46*, 381-471.

(9) Ma, L.; Wang, Q.; Islam, S. M.; Liu, Y.; Ma, S.; Kanatzidis, M. G. Highly Selective and Efficient Removal of Heavy Metals by Layered Double Hydroxide Intercalated with the MoS_4^{2-} Ion. *J. Am. Chem. Soc.* **2016**, *138* (8), 2858–2866.

(10) Ma, S.; Chen, Q.; Li, H.; Wang, P.; Islam, S. M.; Gu, Q.; Yang, X.; Kanatzidis, M. G. Highly Selective and Efficient Heavy Metal Capture with Polysulfide Intercalated Layered Double Hydroxides. *J. Mater. Chem. A* **2014**, *2* (26), 10280–10289.

(11) Li, J. R.; Wang, X.; Yuan, B.; Fu, M. L.; Cui, H. J. Robust Removal of Heavy Metals from Water by Intercalation Chalcogenide $[CH_3NH_3]_{2x}Mn_xSn_{3-X}S_6\cdot0.5H_2O$. *Appl. Surf. Sci.* **2014**, *320*, 112–119.

(12) Pournara, A. D.; Bika, C. G.; Chen, X.; Lazarides, T.; Kaziannis, S.; Feng, P.; Manos, M. J. A Bifunctional Robust Metal Sulfide with Highly Selective Capture of Pb^{2+}ions

and Luminescence Sensing Ability for Heavy Metals in Aqueous Media. *Inorg. Chem. Front.* **2021**, *8*, 4052-4061.

(13) Fu, W.; Ji, G.; Chen, H.; Yang, S.; Yang, H.; Guo, B.; Huang, Z. Engineering Anion Resin Based Amorphous Molybdenum Sulphide Composite for Treatment of Authentic Acid Mine Drainage. *J. Environ. Chem. Eng.* **2020**, *8* (5), 104072.

(14) Lou, J.; Fu, Q.; Yu, L.; Yuan, H.; Zhao, J.; Wang, L.; Shi, D.; Mo, C.; Luo, J. Highly Effective Removal of Pb^{2+} from Wastewater by Nickel-Based Metal Organic Framework. *J. Solid State Chem.* **2022**, *315*, 123535.

(15) Rathee, G.; Kohli, S.; Awasthi, A.; Singh, N.; Chandra, R. MoS_4^{2-}Intercalated NiFeTi LDH as an Efficient and Selective Adsorbent for Elimination of Heavy Metals. *RSC Adv.* **2020**, *10* (33), 19371–19381.

(16) Li, F.; Wang, X.; Yuan, T.; Sun, R. A Lignosulfonate-Modified Graphene Hydrogel with Ultrahigh Adsorption Capacity for Pb(II) Removal. *J. Mater. Chem. A* **2016**, *4* (30), 11888–11896.

(17) Tan, L.; Liu, Y.; Meng, F.; Wu, P.; Xia, Y.; Tang, Y. 3D Hierarchical Defect-Rich C@MoS_2 nanosheet Arrays Developed on Montmorillonite with Enhanced Performance in Pb(II) Removal. *Environ. Sci. Nano* **2020**, *7* (10), 3088–3099.

(18) Wang, Z.; Zhang, J.; Wen, T.; Liu, X.; Wang, Y.; Yang, H.; Sun, J.; Feng, J.; Dong, S.; Sun, J. Highly Effective Remediation of Pb(II) and Hg(II) Contaminated Wastewater and Soil by Flower-like Magnetic MoS_2 Nanohybrid. *Sci. Total Environ.* **2020**, *699*, 134341**.**

(19) Pournara, A. D.; Rapti, S.; Lazarides, T.; Manos, M. J. A Dithiocarbamate-Functionalized Zr^{4+}MOF with Exceptional Capability for Sorption of Pb^{2+} in Aqueous Media. *J. Environ. Chem. Eng.* **2021**, *9* (4), 105474.

(20) Nie, J.; Chandra Roy, S.; Dhami, S.; Islam, T.; Amin, R.; Zhu, X.; Taylor-Pashow, K.; Han, F. X.; Islam, S. M. K-Co-Mo-S_x Chalcogel: High-Capacity Removal of Pb^{2+} and Ag^+ and the Underlying Mechanisms. *J. Mater. Chem. A* **2024**, 12, 30063-30072.

(21) Rathore, E.; Pal, P.; Biswas, K. Layered Metal Chalcophosphate (K-MPS-1) for Efficient, Selective, and Ppb Level Sequestration of Pb from Water. *J. Phys. Chem. C* **2017**, *121* (14), 7959–7966.

(22) Pournara, A. D.; Margariti, A.; Tarlas, G. D.; Kourtelaris, A.; Petkov, V.; Kokkinos, C.; Economou, A.; Papaefstathiou, G. S.; Manos, M. J. A Ca^{2+} MOF Combining Highly Efficient Sorption and Capability for Voltammetric Determination of Heavy Metal Ions in Aqueous Media. *J. Mater. Chem. A* **2019**, *7* (25), 15432–15443.

(23) Khitous, M.; Trari, M. Experimental Study and Modeling of Pb^{2+} Sorption by Calcined Ni/Mo Hydrotalcite in a Fixed-Bed Column. *Chem. Eng. Commun.* **2024**, *212*, 329–344.

(24) Lakshmipathy, R.; Balaji, G. L.; Rico, I. L. R. Removal of Pb^{2+}Ions by ZSM-5/AC Composite in a Fixed-Bed Bench Scale System. *Ads. Sci. Technol.* **2021**, *2021*, 2013259.

(25) Lv, L.; Wang, K.; Zhao, X. S. Effect of Operating Conditions on the Removal of Pb^{2+} by Microporous Titanosilicate ETS-10 in a Fixed-Bed Column. *J. Colloid Interface Sci.* **2007**, *305* (2), 218–225.
